# Haplotype‐resolved genome and pan‐genome graphs reveal the impacts of structural variation on functional genome and feather colors in chickens

**DOI:** 10.1002/imo2.70027

**Published:** 2025-05-24

**Authors:** Lihong Gu, Chen Peng, Anhong Chen, Kaiyu Chen, Xinli Zheng, Dongyou Yu, Zhengguang Wang, Lingzhao Fang, George E. Liu, Pengju Zhao

**Affiliations:** ^1^ Institute of Animal Science & Veterinary Medicine Hainan Academy of Agricultural Sciences Haikou China; ^2^ Hainan Institute Zhejiang University, Yongyou Industry Park Yazhou Bay Sci‐Tech City Sanya China; ^3^ College of Animal Sciences Zhejiang University Hangzhou Zhejiang China; ^4^ Center for Quantitative Genetics and Genomics Aarhus University Aarhus Denmark; ^5^ Animal Genomics and Improvement Laboratory, Beltsville Agricultural Research Center, Agricultural Research Service USDA Beltsville Maryland USA

**Keywords:** chicken, functional genome, haplotype‐resolved assembly, pan‐genome graph, structural variation

## Abstract

Structural variation (SV) refers to a wide range of genetic variations that significantly affect genome biology and complex phenotypes. However, the population‐level SV landscape and its functional impacts on chickens are largely unknown. Here, we generated haplotype‐resolved genome assemblies for the Chinese Wenchang chicken and then integrated them with another 29 assemblies and 354 genome resequencing data to construct a pan‐genome graph for SV genotyping. We detected 185,205 high‐confidence SVs and found that one‐third of them were derived from homology‐based and transposable element (TE) insertion‐based mutational mechanisms. By examining the ChickenGTEx resource, we discovered 1728 SVs associated with molecular phenotypes (e.g., gene expression and alternative splicing), including a 2.7‐kb insertion in the exon of the *EEF1A2* gene related to egg‐laying rates, which showed a significant difference in frequency between broilers and layers. Additionally, we identified a lncRNA gene with a Variable Number of Tandem Repeats (VNTR)‐mediated SV influencing white feathers in Wenchang chicken due to gene flow from white layers. Overall, our study provides a valuable resource for chicken genetics and genomics and sheds light on the SV landscape in chickens as well as its potential contributions to genome structure, gene regulation, and complex traits.

## Highlights

- Pan‐genome graph construction integrates 30 haplotype‐resolved assemblies and 354 resequenced genomes, identifying 185,205 structural variations (SVs) and revealing transposable element (TE)/homology‐driven SV origins in chickens.
- Population‐level SV dynamics highlight broiler‐layer divergence and gene flow shaping trait‐associated SVs, bridging structural variation with avian domestication.
- SV‐phenotype associations uncover 1728 SVs linked to gene regulation and a VNTR‐mediated lncRNA SV driving white feather pigmentation.

## Graphical abstract

This study built a Wenchang chicken haplotype genome and integrated it with 29 others to create a chicken pangenome atlas. Analysis of 354 chickens revealed 185,205 structural variations (SVs), with one‐third derived from homology‐based and transposable elements. We found 1728 SVs linked to traits, including an *EEF1A2* insertion affecting egg‐laying rates and a VNTR‐mediated SV influencing white feathers. These findings advance chicken genetics research and demonstrate SVs' importance in genomics.

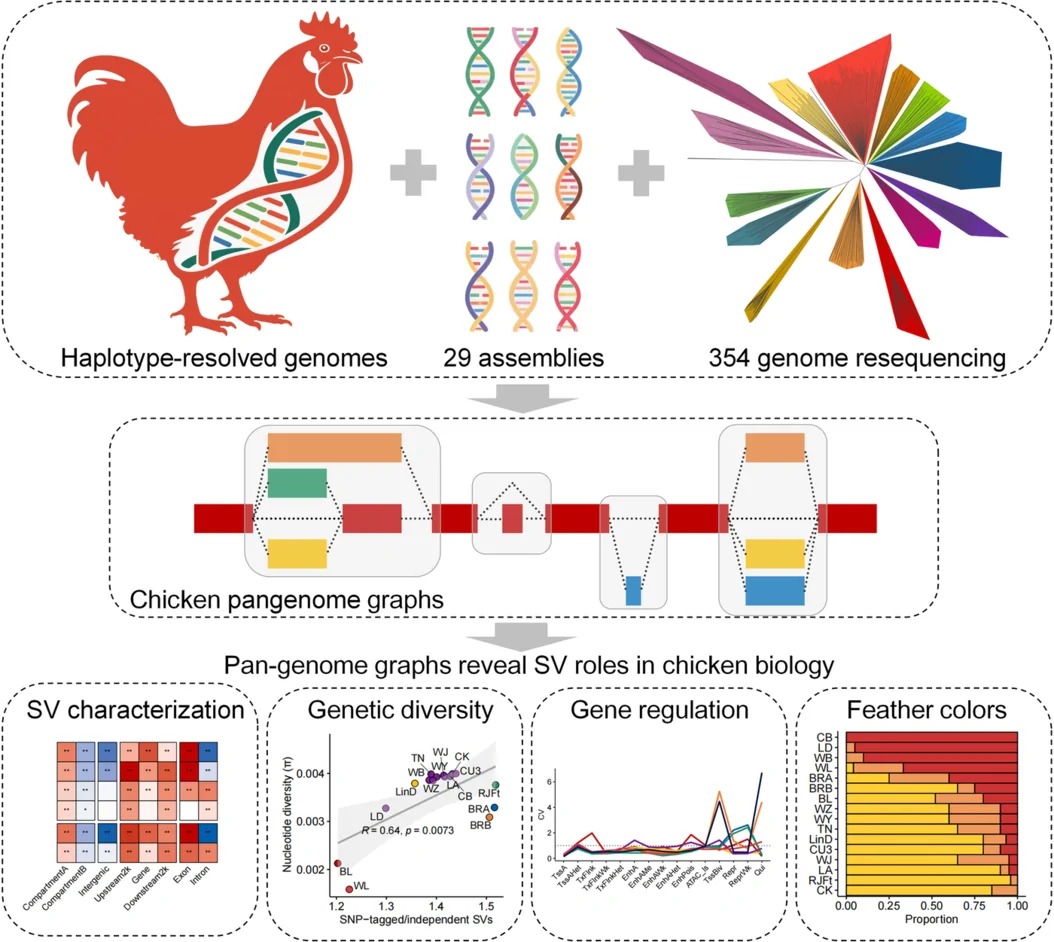

## 1 INTRODUCTION

Chickens have significant economic value in agriculture and are often used as a model for studying developmental biology and genetics [1, 2]. Domestic chickens originally derived from the red jungle fowl (RJF) subspecies *Gallus gallus* spadiceus, undergo both natural and human‐mediated selection, leading to their distinct behavioral, morphological, and reproductive traits, particularly in meat and egg production [3, 4]. Wenchang chicken is a traditional yellow‐feathered breed and the only indigenous chicken breed from Hainan Province listed in Animal Genetic Resources in China (Poultry) [5]. Hainan, being the southernmost province of China and an island, offers a unique local adaptation for Wenchang chickens compared to other yellow‐feathered breeds. It shows genetic differences of varying degrees from the Lindian chicken of northern China, RJF, commercial broilers, and layers, particularly in aspects like meat quality and fertility [6, 7]. Additionally, Wenchang chickens have been selectively bred in local breeding farms for a certain period of time, resulting in the cultivation of substrains that can serve as ideal genetic materials [5].

Structural variations (SVs) are a diverse class of genetic variations that are at least 50 base pairs in size. They can alter the genome through duplications, deletions, transpositions, and inversions of sequences [8, 9]. Although SVs are relatively rare compared to single‐nucleotide polymorphisms (SNPs) and small insertions and deletions (InDels, 1–50 bp), their size and diversity mean that SVs have significant functional impacts on regulatory elements and protein‐coding genes [10]. Previous studies have suggested that SVs play an important role in regulating gene expression and complex traits/diseases in human and livestock populations [11, 12, 13]. Notable examples in chicken genetics include: a 127.4‐kb duplication upstream of *EDN3* that results in hyperpigmentation [14]; an 8.3‐kb deletion upstream of the *SOX10* transcription start site that produces a dark brown phenotype [15]; and a 7.4‐Mb inversion proximal to *MNR2* that influences comb development [16].

Although SVs have been established as a fundamental source of genetic variation in chickens [17], our understanding of their genomic properties and functional implications remains limited. This is primarily due to technical constraints in short‐read sequencing technology, specifically: 1) Challenges in precise and efficient breakpoint identification across large populations [18]; and 2) difficulties in obtaining comprehensive insertion sequences for large genomic fragments [19]. Recent advances in long‐read sequencing technologies have enabled the generation of high‐quality assemblies [20]. Leveraging multiple high‐quality assemblies, graph‐based pan‐genomes facilitated accurate genotype profiling of SVs at a population level [21, 22, 23, 24]. Current poultry pangenome research offers valuable insights into avian evolution, body weight, and body size [25, 26, 27]. As a result, researchers can now study previously hidden inherited traits—both molecular and complex—and identify their associated SVs through methods like SV‐based genome‐wide association studies (GWAS) and expression quantitative trait loci analyses [28, 29, 30].

In this study, we first constructed haplotype‐resolved assemblies for the Wenchang chicken and then integrated them with another 29 existing genome assemblies to create a comprehensive pan‐genome graph in chickens. Leveraging this pan‐genome graph, we performed high‐quality SV genotyping on 354 whole‐genome sequenced chickens. We systematically investigated the molecular mechanisms of SV formation and examined their impacts on the genome structure of chickens. By examining resources from the Chicken FAANG and ChickenGTEx projects [31, 32], we found that SVs tended to occur frequently in regulatory elements and exhibited tissue‐specific characteristics. Based on linkage disequilibrium (LD) analysis, we identified 1728 SVs that showed potential cis‐regulatory activity on molecular phenotypes (e.g., gene expression and alternative splicing), with significant differences in frequency between broilers and layers. Additionally, we detected a lncRNA with a VNTR‐mediated SV [33, 34], which is significantly associated with white feathers, potentially due to the gene flow from the white layer to Wenchang chickens. Overall, our study provides an invaluable resource for chicken genetics and genomics, as well as highlights the importance of SVs in studying genome structure, gene regulation, and complex traits.

## 2 RESULTS

### A haplotype‐resolved assembly of Wenchang chicken and its annotation

**FIGURE 1:**
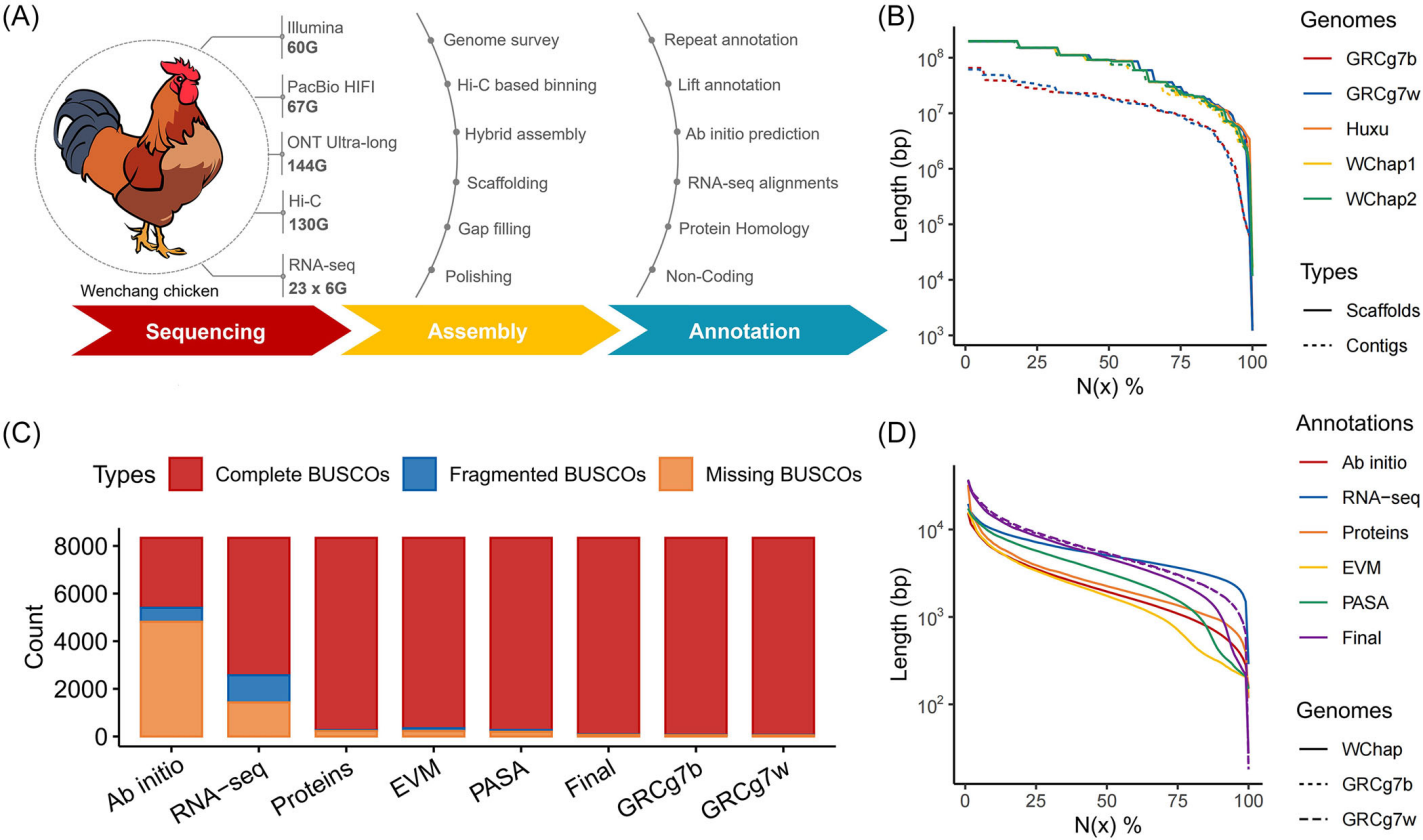
Assembly and annotation of the Wenchang chicken genome. (A) The overview of the sequencing information, assembly strategy, and annotation methods for the Wenchang chicken genome. (B) A cumulative contiguity plot is used to compare the assembly contiguity of the chicken reference genomes (layer ‐ GRCg7w and broiler ‐ GRCg7b), Huxu, and our genomes at the scaffold and contig levels. (C) The BUSCO evaluation was performed for the protein‐coding annotations using different annotation methods and genomes. EVM: all predicted evidence was integrated as nonredundant gene annotation by EvidenceModeler. PASA: Updating gene models for the identification of alternative splice sites and UTR annotation. (D) A cumulative contiguity plot is used to compare the contiguity of genome annotation across different annotation methods and genomes.

To assemble the Wenchang chicken genome, we generated 66.3× Pacific Biosciences (PacBio) HiFi, 142.6× Oxford Nanopore Technologies (ONT) ultra‐long, 59.4× Illumina paired‐end, and 130 Gb Hi‐C sequencing data (Figure 1A). The estimated genome size, based on k‐mer statistics, is 1.01 Gb, with heterozygosity of 0.57% and 8.44% of the sequences being repetitive (Figure S1). To achieve a complete haplotype resolution, we divided all reads into two read bins using Hi‐C‐based binning genomes [35, 36]. Utilizing Verkko, we generated haplotype‐resolved contigs by combining ultra‐long ONT reads and HiFi reads through hybrid assembly approaches [37]. The pre‐binned haplotype reads were used to accurately infer haplotype walks for Verkko. After assembling each haplotype, the contigs were scaffolded to the chromosome level using Hi‐C data. Then, gap‐closing was performed through ONT‐based assembly, and polishing was carried out to correct errors based on pre‐binned HiFi and Illumina reads (Figures 1A and S2).

The two final haplotype‐resolved assemblies, WChap1 and WChap2, consist of 73 and 65 scaffolds, with total lengths of 1.11 and 1.10 Gb, respectively. These assemblies received almost complete support from at least two sequencing technologies for spanning (98.47% and 97.53% reliable blocks, respectively, Figure S3). They achieved a high level of contig anchoring, with 97.60% and 98.76% of the contig length mapped to 39 autosomes, Z sex chromosomes, and a mitochondrial genome, respectively. Of these, 21 and 25 chromosomes are available with telomeres at both ends (Table S1), respectively. Additionally, both new assemblies exhibit high consensus quality metrics (QV = 32.90 and 32.73), alongside enhanced contiguity (Figure 1B) and completeness (Table 1). These metrics notably exceed those of the current reference genome (GRCg7b and GRCg7w) and align closely with the benchmarks established by the Huxu chicken T2T genome [38].

**TABLE 1:** Quality statistics of the chicken genomes.

Statistics	WChap1^a^	WChap2^a^	Huxu^b^	GRCg7b^c^	GRCg7w^c^
Genome size (bp)	1,105,035,104	1,097,851,144	1,100,928,095	1,053,332,251	1,050,511,239
Scaffolds number	73	65	41	214	276
Scaffold N50 (bp)	91,553,107	91,836,423	91,364,047	90,861,225	90,563,926
Max scaffold (bp)	200,098,415	200,008,622	200,044,509	196,449,156	196,242,913
Min scaffold (bp)	16,516	11,784	16,785	1437	1219
Contig number	80	71	55	677	685
Contig N50 (bp)	75,248,722	91,836,423	91,364,047	18,834,961	17,744,542
GC content (%)	42.61	42.55	42.64	42.20	42.16
Gap number	7	6	14	463	409
Telomere number	51	51	52	30	17
BUSCO completeness (%)	96.6	96.7	96.7	96.7	96.8
Complete BUSCOs	8058	8061	8066	8062	8073
Missing BUSCOs	219	218	211	212	203
Fragmented BUSCOs	61	59	61	64	62

^a^ Two haplotype‐resolved assemblies, WChap1 and WChap2.

^b^ Huxu chicken genome.

^c^ Chicken reference genomes, GRCg7b and GRCg7w.

In the genome annotation for WChap1 and WChap2, 10.59% and 10.58% of these genomes were identified as transposable elements (TEs), with the most prevalent subclass being the LINE/CR1 lineage, accounting for 8.79% and 8.91% of the genomes (Table S2), respectively. To predict protein‐coding genes, a customized pipeline was used to integrate lift annotation, ab initio gene prediction, RNA‐Seq assemblies from 23 tissues, and protein homology alignment. EvidenceModeler consolidates all evidence for precise gene prediction, while the PASA pipeline combines gene models to identify alternative splice sites and annotate untranslated region (UTR) (Figure S4). This process resulted in the identification of 17,652 and 17,651 protein‐coding genes, with an average of 4.04 and 4.05 transcripts per gene, respectively. Compared to the single method, this integrative approach yielded a higher quantity of genome annotation, which was comparable to the reference annotation (Figure 1C,D, Table S3). In summary, these results demonstrate the high quality of the haplotype‐resolved assemblies and their annotations.

### Building pangenome graphs and genotyping structural variations

**FIGURE 2:**
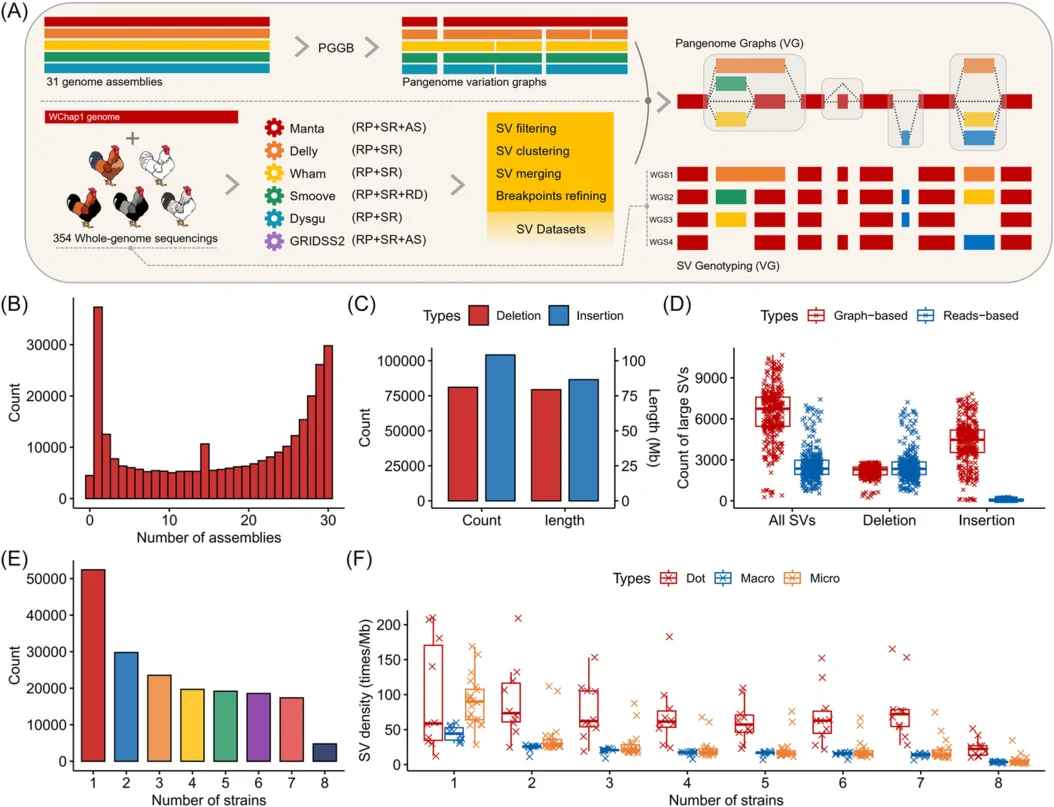
Genotypes of structural variants based on pangenome graphs. (A) The pipeline for constructing pangenome graphs based on assembly‐ and reads‐based methods and genotyping of structural variations (SVs). (B) The counts of the “bubble” subgraphs supported by a varying number of assemblies. (C) The counts and lengths of all 185,205 genotyped SVs. (D) Comparison of graph‐based and read‐based SV counts. (E) The counts of SVs supported by different numbers of strains. (F) The distribution density of SVs in different chromosome types. Chromosome types can be split into macro, micro, and dot chromosomes based on their length. *X*‐axis: SVs supported by different numbers of strains.

We utilized the PanGenome Graph Builder (Pggb) pipeline to construct pangenome variation graphs from 31 assemblies of 27 samples for each chromosome [39] (Figure 2A, Table S4). Using the WChap1 assembly as the foundation of the graph structure, we identified a total of 302,378 large “bubble” subgraphs corresponding to nonoverlapping variant sites (≥50 bp) through graph decomposition. Most of these variant sites can be classified as putative ancestral polymorphisms, with an average sequence change of 0.81 kb (Figure 2B). In addition, to enhance the study of polymorphic SVs in different populations of Wenchang chickens, we employed six tools that utilize at least two detection signals (RP, read pairs; SR, split reads; RD, read depth; AS, assembly) to identify SVs (Figure 2A). After filtering these initial predictions and SV breakpoints that were detected less than three times, we obtained a total of 876,127 read‐based SVs.

Using the vg toolkit [40], we constructed the final chicken pangenome graphs based on the aforementioned SVs, with the WChap1 assembly serving as the backbone of the graph structure (Figure 2A). A total of 654,317 nonredundant SVs were incorporated into the graph as final “bubble” subgraphs, encompassing 187.23 Mb of genomic deletions and 265.16 Mb of non‐reference sequences (≥50 bp). Using vg giraffe [41] coupled with this pangenome graph, we genotyped the biallelic SVs in 354 chickens from 8 strains (17 populations), with an average coverage of 14.8× of Illumina short reads. This approach allowed the genotyping of 185,205 SVs (28.31%) of all subgraphs with a calling rate higher than 0.3 and *a minor* allele count greater than 3. Among these SVs, insertions were more abundant and larger than deletions, with the largest insertion being 244.7 kb (Figures 2C and S5). On average, we identified 6822 large SVs (>200 bp) for each sample using pangenome graphs. This is almost twice the number of large SVs per sample detected through the read‐based strategy (supported by at least two tools) on the current linear chicken reference assembly, highlighting the effectiveness of pangenome graphs in detecting large SVs, especially insertions (Figure 2D).

The majority of SVs were present at low frequencies. Approximately 23.49% of SVs can be classified as rare, as they were found in less than 1% of all the samples. Furthermore, 28.30% of SVs were identified in only one strain, while 2.57% were shared across all chicken strains (Figure 2E). These SVs, which were only observed in single strains, showed a preference for microchromosomes and were less abundant in macrochromosomes (90.66 times/Mb vs. 40.06 times/Mb) (Figure 2F). Interestingly, regardless of the frequency of SVs, dot chromosomes exhibit higher breakpoint rates compared to other chromosomes. This bias may be associated with the distinct recombination rates of the chicken dot chromosomes [42].

### Characterization and molecular formation of structural variations

**FIGURE 3:**
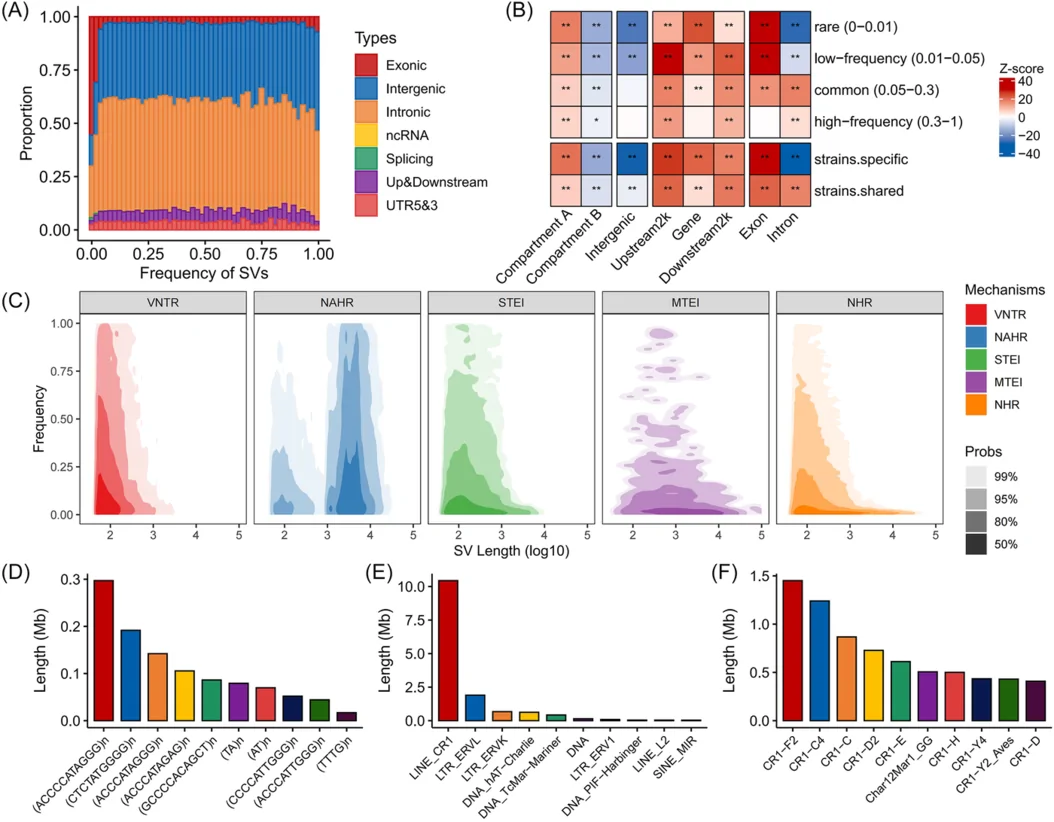
Characteristics of structural variation in chicken. (A) The distribution of structural variation (SV) with different frequencies across different functional regions. (B) A heatmap shows the levels of over‐ and under‐representations for different SV catalogs in various functional regions. Statistical significance for the enrichment was indicated as follows: *p < 0.05, **p < 0.01, ***p < 0.001. (C) Density plots illustrate the distribution in length and frequency of SV from different formation mechanisms. The probability values (Probs) indicate the density of different SV types. (D) Bar plots show the genomic lengths of various tandem repeats. (E) Bar plots show the genomic lengths of different TE‐related SV sequences. (F) Bar plots show the genomic lengths of different LINE CR1‐related SV sequences.

The common SVs (with frequency ≥ 5%) were mainly located in intergenic and intronic regions, accounting for 69.22% of the total, while nearly one‐third of rare and low‐frequency SVs (with frequency < 5%) were found in coding and regulatory regions (Figure 3A). In general, SVs with different frequencies showed similar enrichments across various functional features of the genome, except for introns (Figure 3B). Of note, SVs were more enriched in active genomic regions compared to inactive regions (Figure 3B). For example, SVs were more likely to be found in the A compartments rather than B compartments, as well as in gene bodies and their upstream and downstream regions, rather than intergenic regions. Notably, we observed a high enrichment of SVs with lower frequencies in exons, particularly rare SVs and strain‐specific SVs. Even so, there were still 256 protein‐coding genes whose exons were affected by high‐frequency SVs. Some of these genes have been proven to be promising candidate genes in biological processes such as body mass and growth (e.g., the *YEATS4* gene [43]), production performance (e.g., the *COX5A* gene [44]), and immunity development (e.g., the *BG1* gene [45]).

Next, we examined the formation mechanisms of SVs based on their unambiguous breakpoint locations using the BreakSeq. 2 algorithm [46]. We discovered that 30% of SVs resulted from homology‐based and TE insertion‐based mechanisms, including 10.66% from nonallelic homologous recombination (NAHR), 10.72% from single TE insertion (STEI), 7.95% from variable number of tandem repeats (VNTRs), and 0.3% from multiple TE insertion (MTEI). The remaining nonhomologous recombination SV events (NHR) may be linked to nonhomologous end‐joining or microhomology‐mediated break‐induced replication [46]. We observed that homology‐based SVs occur with higher frequency than others (Figure 3C). The size of SVs mediated by NAHR typically ranges from 1000 to 10,000 bp. VNTR‐mediated SVs typically involved expansions and contractions of short repeat units that were 2–11 bp long, such as the (ACCCCATAGGG)n satellite repeat (Figure 3D). This could be due to replication slippage‐mediated expansion and contraction like fork stalling and template switching [47]. Notably, unlike in mammals, as TEs account for only 10% of the genome, TEs may not be the primary driver of SVs in the chicken genome. Furthermore, our analysis shows that approximately 90% of TE‐mediated SVs can be attributed to the LINE/CR1 family (Figure 3E), which consists of multiple subfamilies involving both older (CR1‐C4) and younger subfamilies (CR1‐F2) [48] (Figure 3F). These subfamilies of the CR1 family make comparable contributions to the overall regions of SV sequences, but the sequences from the CR1 subfamilies are primarily concentrated in the 3' ends (Figure S6).

### Structural variants revealed novel population structure of chicken populations

**FIGURE 4:**
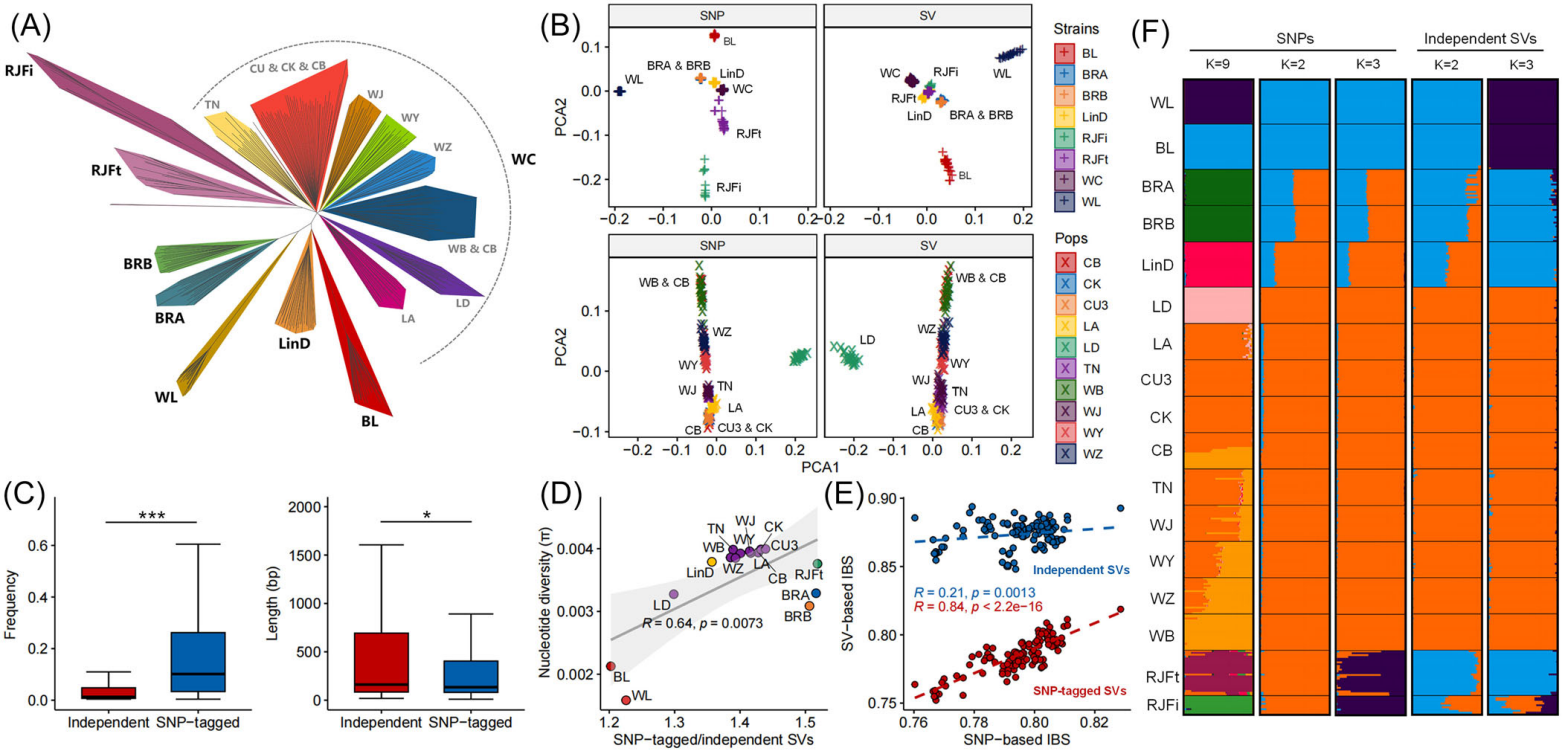
Roles of structural variation in chicken genetic structure. (A) Phylogenetic tree based on single‐nucleotide polymorphisms (SNPs) for 354 chickens. (B) PCA plots display the genetic relationship among 354 chickens based on SNPs and structural variations (SVs), respectively. The study encompasses several distinct populations: White layer (WL), Brown layer (BL), BRA and BRB Broiler lines (BRs), Lindian chicken (LinD), Wenchang chicken (WC), and red jungle fowl specimens from Thailand (RJFt) and India (RJFi). Within the Wenchang chicken group, ten subpopulations are designated according to their corresponding breeding facility identifiers. (C) The difference in frequencies and lengths between independent SVs and SNP‐tagged SVs. The two‐sided Wilcoxon test was used to assess statistical significance. Statistical significance was indicated as follows: *p < 0.05, **p < 0.01, ***p < 0.001. (D) The relationship between SV categorization and genetic diversity. The *x*‐axis represents the ratio of SNP‐tagged SVs to independent SVs, while the *y*‐axis shows SNP‐based nucleotide diversity (*π*). (E) The relationship between SNP‐based and SV‐based identity by state (IBS) distance. Red indicates SNP‐tagged SVs, while blue indicates independent SVs. The correlation coefficient (*R*) quantifies the relationship between SNP‐based and SV‐based IBS distance, while the associated *p‐*value demonstrates the correlation's statistical significance. (F) Population structure based on SNPs and SVs for 354 chickens when K was 2, 3, and 9, respectively.

A phylogenetic tree constructed for 354 chickens using SNPs clearly separated WL (White layer), BL (Brown layer), BRs (BRA and BRB Broiler lines), LinD (Lindian chicken), WC (Wenchang chicken), RJFt (red jungle fowl from Thailand), and RJFi (red jungle fowl from India) (Figure 4A, Tables S5–6). In addition, these samples represent a variety of chicken strains with different levels of genetic diversity and LD decay (Figure S7), making them suitable for evaluating the influence of SVs on population structure. We thus estimated genetic segregations among these chicken strains using SVs and compared them with those derived from SNPs above (Figure 4B). Principal component analysis revealed similar patterns of separation among chicken strains between SVs and SNPs, except for the RJFi and RJFt strain. Both the WL and BL strains demonstrated a clear distinction from other strains, as indicated by their distinct SV frequency spectra (Figure S8). This differentiation likely resulted from the distinct adaptations of specialized egg‐laying birds (layers) and fast‐growing meat birds (broilers) during their respective domestication processes [7]. Similarly, we observed a consistent genetic separation among the 10 distinct populations of the WC chicken strain, regardless of the type of genetic variation (Figure 4B). This pattern of similar separation could be due to the LD between SNP and SV.

To avoid the impact of LD, we extracted 107,235 SVs (57.90% of total SVs) not in LD with nearby SNPs (*R* ^2^ < 0.2). Compared to SVs tagged by SNPs (*R* ^2^ > 0.2), these independent SVs showed significantly lower allele frequencies and longer sequence lengths, particularly those longer than 5 kb (Figure 4C). For each strain or population, the number of SNP‐tagged SVs was higher than that of independent SVs, and the ratio of SNP‐tagged SVs to independent SVs showed a positive correlation with the population genetic diversity (Figure 4D). Furthermore, we calculated the identity by state (IBS) distance between individuals using both SNP‐tagged and independent SVs to compare the differences in their contributions to relatedness. We observed that IBS distances calculated by SNP‐tagged SVs were similar to those by SNPs, while the IBS distances of independent SVs were higher than those obtained from SNPs (Figure 4E).

Population admixture inference was conducted using various genetic variations to investigate the potential hidden effects of independent SVs on the population structure (Figure 4F). The primary cluster, based on SNPs, revealed genetic segregations that align with the phylogenetic tree at the lowest cross‐validation error value, corresponding to *K* = 9. However, for independent SVs, the lowest cross‐validation error value was observed when *K* was 2. Red Junglefowls (RJFs) displayed a closer genetic relationship with commercial broilers and layers than with WC chickens. When *K* was 3, commercial layers formed a distinct cluster separating from other strains. These results suggest that independent SVs are more likely to be specific to certain strains and may harbor a hidden genetic architecture that differs from that of SNPs.

### Structural variations contribute to gene regulation

We evaluated the relationship between regulatory elements and the accumulation of SVs, which may result in changes in gene dosage frequency [49]. As part of the Functional Annotation of Animal Genomes (FAANG) project [31], seven types of chicken regulatory elements involving 15 distinct chromatin states were identified in 23 chicken tissues. On average, 96.91% of these regions can be lifted over from the galGal6 to WChap1 genome. Meanwhile, eight categories of SVs with different frequency ranges were defined for the enrichment analysis of SVs in these functional regions.

**FIGURE 5:**
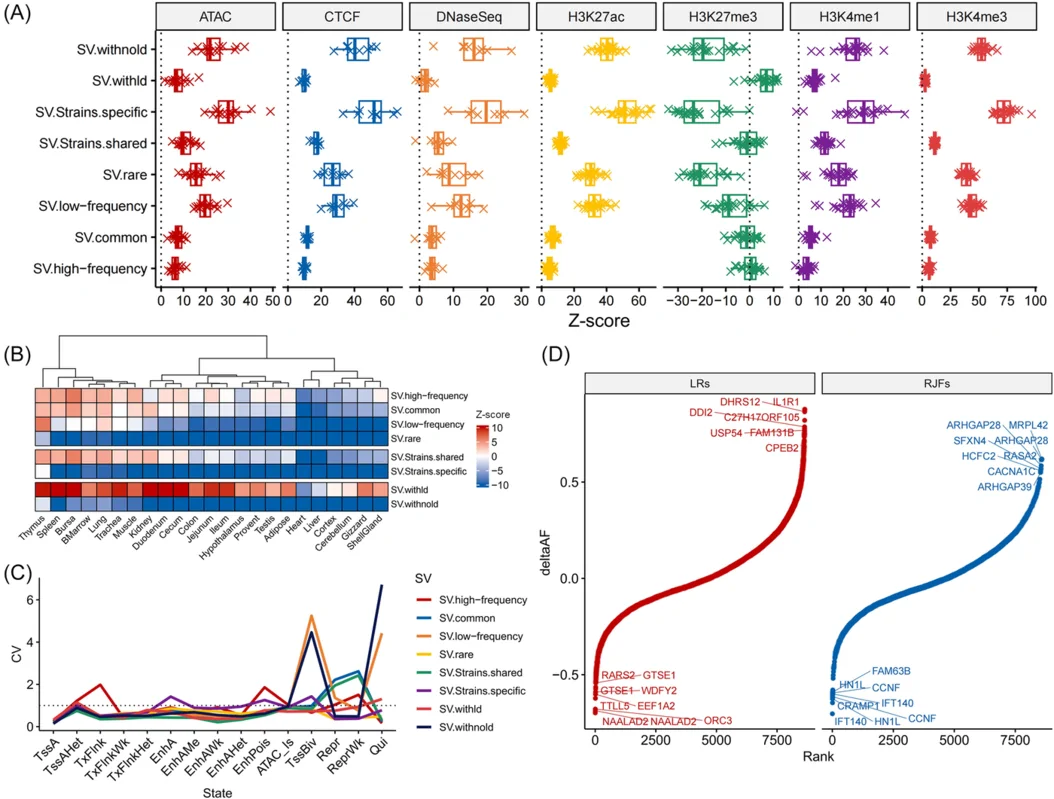
Association of structural variants with putative regulatory regions. (A) Boxplots display the enrichment levels of structural variations (SVs) of different types in various regulatory regions. SVs were categorized as: rare (frequency ≤ 0.01), low‐frequency (0.01 < frequency ≤ 0.05), common (0.05 < frequency ≤ 0.3), or high‐frequency (0.3 < frequency). They were also classified as strain‐specific (present in a single strain) or strain‐shared (present in multiple strains), and as either withld (*R*² ≤ 0.2) or withnold (*R*² > 0.2) in the categories. (B) The heatmap illustrates the distinct over‐ and under‐representation patterns of different SV types overlapping with the H3K27me3 mark. (C) The bar plots show the coefficient of variation of over‐ and under‐representation levels for SVs with different types across 15 chromatin states. (D) Dot plots show the distribution of deltaAF value between layers (LRs) and red jungle fowl (RJFs) to other strains, respectively.

We observed a significant enrichment of SVs on regulatory elements, particularly for SVs in the rare and low frequency, strain‐specific, and independent categories (Figure 5A). In general, the enrichment of SVs in active regulatory elements was consistent across different tissues. Of note, H3K27me3, a histone mark associated with gene repression, was enriched with SVs of high frequency, especially in the immune system (thymus, spleen, bursa, and bone marrow), respiratory system (lung and trachea), and muscle (Figure 5B). We further evaluated the difference in SV enrichments across chromatin states in different tissues using the coefficient of variation (CV). The results showed that enrichment of SVs in most promoters (TssA and TssAHet), enhancers (EnhA, EnhAMe, EnhAWk, EnhAHet, and EnhPois), and TSS‐proximal transcribed regions (TxFlnk, TxFlnkWk, and TxFlnkHet) were consistent among tissues (Figure 5C). However, there were still some tissue‐specific enrichments in these conserved regions. For example, high‐frequency SVs showed high enrichments in promoters (TssAHet) of adipose, cortex, muscle, and hypothalamus tissues, which are potentially relevant to the artificial selection process in chicken breeding.

To investigate the effect of SVs on gene expression, we identified 1,728 SVs (0.93%) having a high LD (*R* ^2^ ≥ 0.6, indicating a strong LD between SVs and SNPs in chickens [50]) with molQTL‐SNPs detected in the ChickenGTEx project [32], referred to as molQTL‐SVs. These molQTL‐SVs showed a higher frequency compared to the entire set of SVs and were likely to affect multiple molecular phenotypes simultaneously (Figure S9). Nearly 80% of the molQTL‐SVs were located within genes, with 85.26% of which were found in the intron regions. For the 85.47% of molQTL‐SVs located in intergenic regions, the distance between the molQTL‐SVs and their potential target genes was less than 5 kb. To test whether these molQTL‐SVs were potentially associated with specific variety‐specific traits, we calculated the delta allele frequency (*delta*AF) for each molQTL‐SV from broilers to commercial layers. We found some genes with a high *delta*AF associated with production performance. For example, a 2.7 kb molQTL insertion found in the exon of the *EEF1A2* gene, with a low *delta*AF of −0.601, has a higher frequency in WC chickens compared to commercial layers (Figure 5D). The expression of *EEF1A2* gene in the ovarian tissues of chickens has been proposed to be associated with egg‐laying rates [51]. Additionally, consistent with a previous study on SNPs [7], molQTL‐SVs related to the *JPT2* (*HN1L*) and *CRAMP1* genes showed significant differences in *delta*AF between RJF and all domestic chickens. For example, a 112 bp molQTL insertion located downstream of the *JPT2* gene, known for its role in embryo development, may be associated with adaptation in domestic chickens [52].

### Impacts of structure variants on chicken feather colors

**FIGURE 6:**
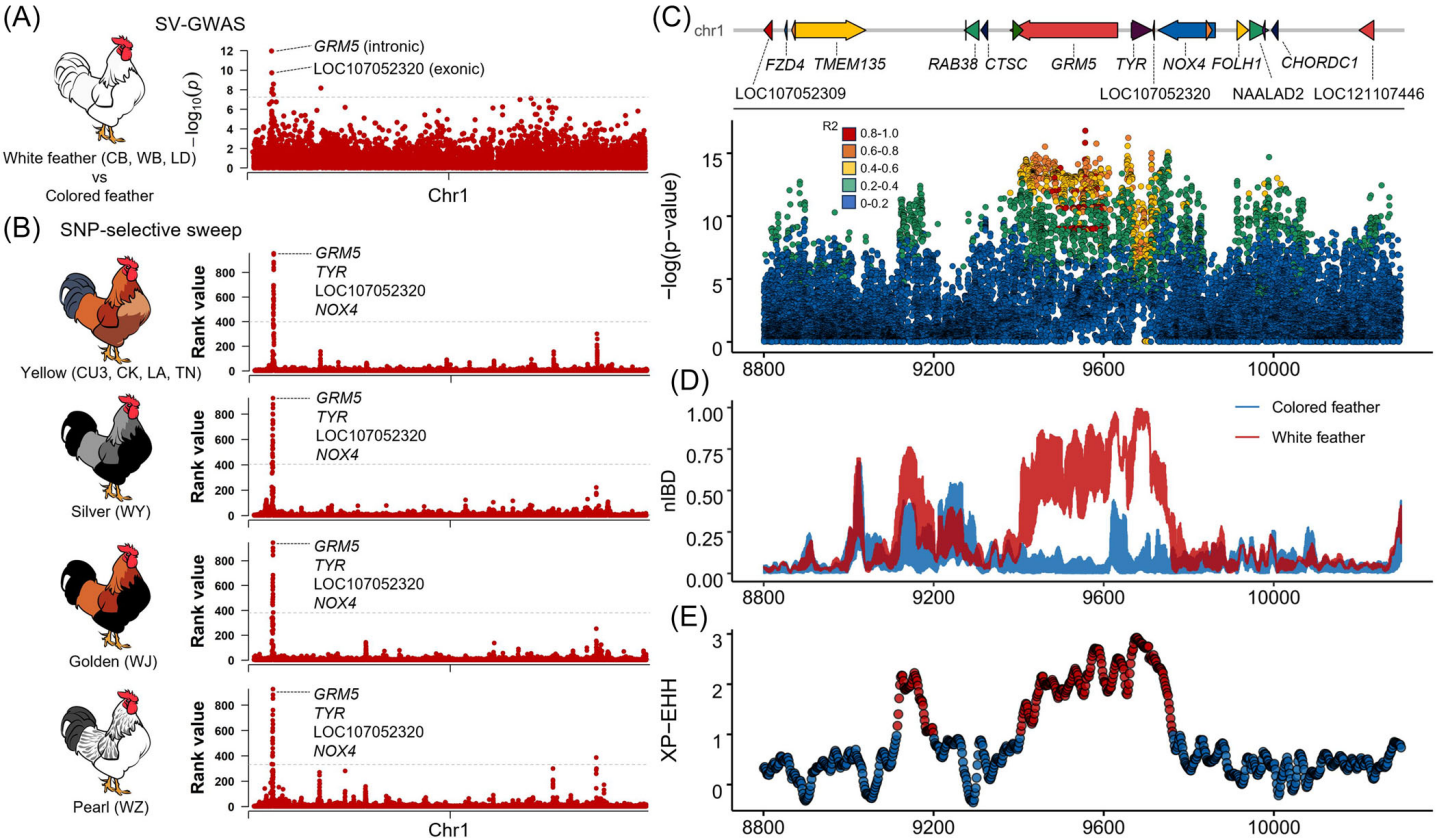
Identification of candidate regions related to feather color. (A) GWAS analysis comparing white and colored feathers using structural variations (SVs). (B) Selective sweeps based on single‐nucleotide polymorphisms (SNPs) between white feathers and various colored feathers were analyzed using rank values. The rank value was calculated by integrating *F*_ST_, XP‐CLR, and XP‐EHH methods. The dotted line shows the threshold for the top 0.1% of rank values. (C) GWAS results for SNP, InDel, and SV in the LD block region between white and colored feathers. The colored dots indicate the LD degree (*R*²) between variants and the lead SNP. (D) Normalized IBD (nIBD) values among chicken groups with white and colored feathers. (E) Dot plot displays the XP‐EHH results in the LD block region between white and colored feathers.

Although more than 10 genes (such as *GRM5*, *PMEL*, and *TYRP1*) are thought to affect chicken feather color, the role of SVs in feather color remains to be explored. In the WC chickens, there are 10 populations with relatively low genetic distance (average *F*_ST_ = 0.035), displaying five distinct types of feather colors, that is, white, yellow, silver, golden, and pearl. This presents an excellent opportunity to investigate the influence of SVs on feather color in chickens. Therefore, we conducted an SV‐based GWAS analysis, which identified 15 SVs significantly associated with the segregation of white and colored feathers (Bonferroni‐corrected *p*‐value ≤ 0.01) (Figure 6A).

**FIGURE 7:**
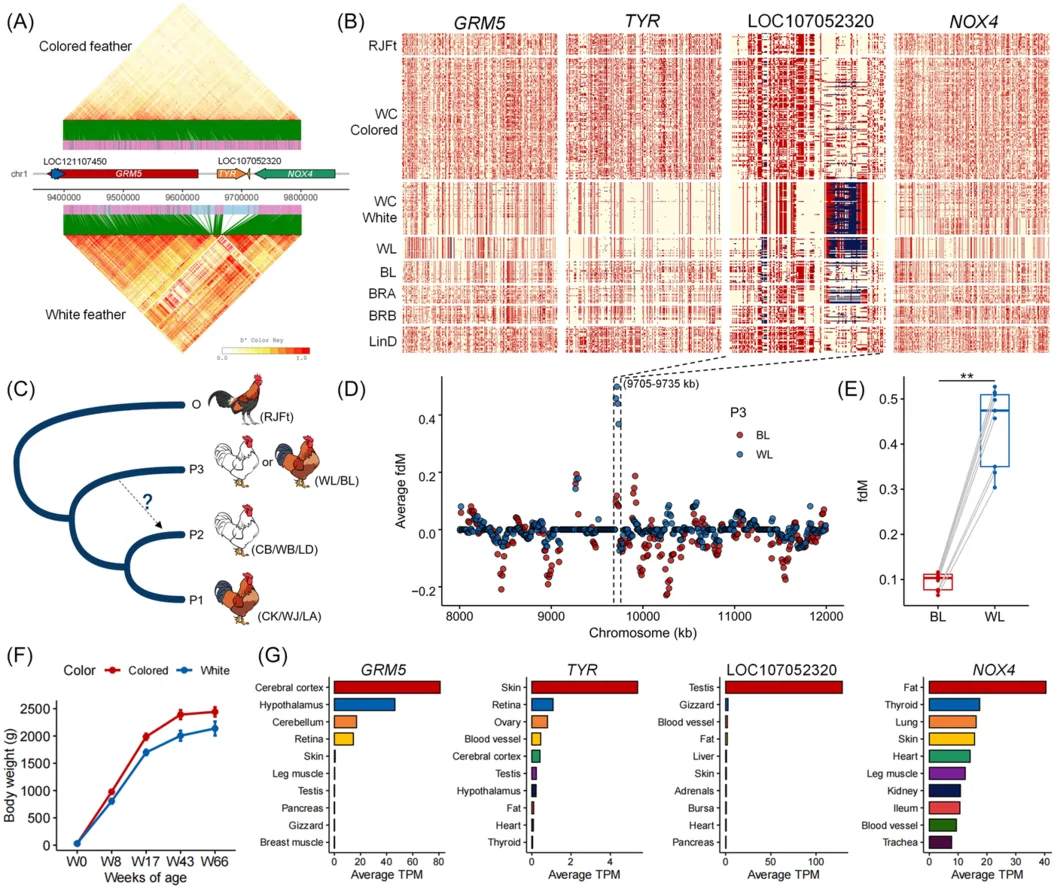
Exploring the role of structural variation in feather color. (A) The LD block heatmap displays the pattern of LD and the haplotype block structure of single‐nucleotide polymorphisms (SNPs) in Wenchang chickens with white and colored feathers. (B) The heatmap displays the SNP genotypes of four genes in the chicken population. Light yellow represents the reference type, red indicates the mutant type, and blue signifies the missing type. (C) The expected model of the D statistic in chickens. The outgroup (O) is represented by RJFt. P1 and P2 are Wenchang chickens with different feather colors. P3 includes the WL and BL. (D) The dot plot shows the ABBA‐BABA statistics in sliding bins. The *x*‐axis denotes the location of sliding bins, while the *y*‐axis indicates the average fdM for each sliding bin. (E) The boxplot represents the fdM distribution for each expected model when P3 is between WL and BL. A two‐sided Wilcoxon test was used to assess statistical significance. Statistical significance was indicated as follows: *p < 0.05, **p < 0.01, ***p < 0.001. (F) Variation in body weight between white and colored feather varieties of Wenchang chickens across different ages. (G) Bar plots display the tissue expression patterns of four genes.

Several genes associated with these significant SVs have been previously identified as potential genes for feather colors. For instance, the *TYR* gene contains a retrovirus insertion that was associated with recessive white feather color in Yeonsan Ogye chickens [53]. The *GRM5* gene is considered a candidate gene for yellow feather pigmentation in Baicheng You chickens [54]. Importantly, regardless of which feather color, these genes can be distinguished based on their rank values (combining *F*_ST_, XP‐EHH, and XP‐CLR) compared to recessive white feathers (Figure 6B). Notably, the genes *GRM5*, *TYR*, LOC107052320, and *NOX4* within the nearly 300 kb region from chr1:9,430,000‐9,740,000, are especially distinctive. The most significant SNP (chr1:9,556,676), with a Bonferroni‐corrected *p*‐value of 3.07 × 10^−10^, was found in the intron of the *GRM5* gene. This SNP was located in a large LD block, which contains numerous significant variants that have an *R* ^2^ value greater than 0.8, such as an InDel (chr1:9,657,777‐9,657,784; GAAAAAAT to G; Bonferroni‐corrected *p*‐value of 1.13 × 10^−9^) upstream of the *TYR* gene (Figure 6C). Furthermore, we assessed the identity‐by‐descent (IBD) for this LD block by calculating the normalized IBD (nIBD) values among chicken groups with white and colored feathers (Figure 6D). Our results showed a higher shared IBD (approximately 80%) among individuals with white feathers, and none among those with colored feathers. This pattern can also be reflected in the high‐frequency difference of long‐range haplotype homozygosity (Figure 6E), with the high LD block only present in the white‐feathered chicken population (Figure 7A).

Upon further scan of the SNP map for these four genes (Figure 7B), we confirmed that this region is under strong selection, especially for the *TYR* and LOC10805230 in white‐feathered WC chickens. It can be speculated that long‐term selection for white feathers resulted in these unique sweep footprints. Importantly, we discovered that white‐feathered WC chickens shared haplotypes with the WL, specifically the same deletion in the exon of LOC10805230. To interpret this, we conducted the ABBA‐BABA statistics to test for potential introgression across chickens with different feather colors [55]. The ABBA‐BABA statistics were performed using the relationship (((P1,P2),P3),O), where the outgroup O was RJFt, P1, and P2 were WC chickens with different feather colors, and P3 selected the WL and BL as the control group (Figure 7C). The results revealed a distinct gene flow event on LOC10805230, ranging from chr1:9,705‐9,735 kb, from WL to white‐feathered WC chickens (Figure 7D). The difference in introgression of WL is observed in WC chickens between white and colored feathers, across all subgroups or populations (Figure 7E). Interestingly, supporting our hypothesis of gene flow from WL to WC chickens, white‐feathered WC chickens showed a lesser trend of weight change with growth compared to those with colored feathers (Figure 7F). Additionally, using the ChickenGTEx atlas, we examined the expression levels of these four genes (Figure 7G). The *GRM5* gene was specific to the nervous system, while the *TYR* gene was highly expressed in pigment‐associated tissues like skin and retina. LOC107052320 was a tissue‐specific lncRNA expressed in the testis, and the *NOX4* gene was ubiquitously expressed but enhanced in fat tissue.

### A novel candidate lncRNA associated with chicken feather colors

**FIGURE 8:**
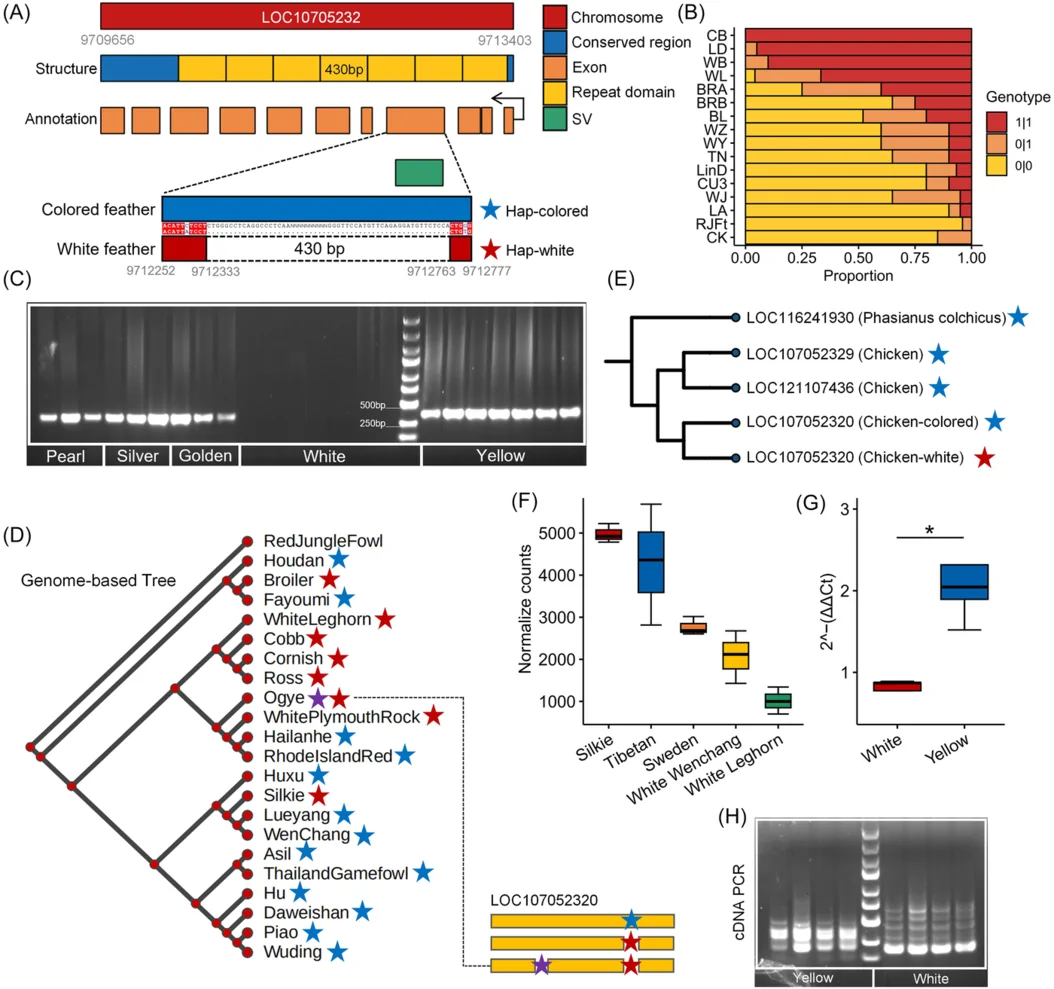
Candidate lncRNA associated with chicken feather color. (A) The structure of the genome and transcript for LOC107052320 and its structural variations (SVs). (B) The proportion of genotypes for the SV within different chicken populations. (C) PCR Validation for SV in different WC chicken Populations. (D) SV haplotypes across 22 chicken assemblies. The yellow bars represent three types of SV haplotypes. (E) SV haplotypes across LOC107052320 and its homologous lncRNAs. (F) Expression levels of LOC107052320 across five chicken strains. (G) qRT‐PCR results for LOC107052320 in WC chickens with white and yellow feathers. A two‐sided Wilcoxon test was used to assess statistical significance. Statistical significance was indicated as follows: *p < 0.05, **p < 0.01, ***p < 0.001. (H) PCR for cDNA of LOC107052320.

We concentrated on a deletion SV in the exon of the LOC107052320 lncRNA locus, which is a novel lncRNA. This variant was identified in regions associated with feather color and could potentially play a significant role in influencing this trait. We thus conducted a structural analysis of its body region and discovered two conserved regions and a 430 bp tandem repeat with 6.79 copies (Figure 8A). The SV was found in exon 4 of LOC107052320, representing the white haplotype. Interestingly, the SV's length was also 430 bp, indicating that the formation mechanism of this SV was likely due to VNTRs.

The SV was nearly fixed in both the white‐feathered WC chicken populations and WL population (Figure 8B). This was further corroborated by the results of the PCR experiments (Figure 8C). In our thorough scan of this SV in 21 domestic chicken genome assemblies, we consistently found the white haplotype in white‐feathered breeds (Figure 8D). The only exception was the Ogye chicken, a breed from Korea with entirely black feathers, but carrying the white haplotype. Interestingly, we observed an additional 430 bp deletion in the LOC107052320 of the Ogye chicken, suggesting the possibility of a third haplotype in this breed (Figure 8D). Next, we examined the SV haplotype of LOC107052320 in relation to its two paralogous lncRNAs in chickens and its orthologous lncRNA in the common pheasant (*Phasianus colchicus*, a colorful and widespread game bird) (Figure 8E). Our findings indicated that the colored haplotype of LOC107052320 was ancestral, while the white one was a derived state.

We evaluated the impact of SV on LOC107052320 expression by analyzing 15 RNA‐seq datasets from testicular tissues. We normalized gene read counts using generalized linear models and assessed the variations in LOC107052320 expression across chickens with different feathers. As expected, chickens with colored feathers showed a higher LOC107052320 expression than those with white feathers (Figure 8F). This was further confirmed by qRT‐PCR results from WC chickens with both colored and white feathers (Figure 8G). Notably, PCR for cDNA of LOC107052320 around this SV breakpoint revealed variations in transcript sequences (Figure 8H), suggesting that the SV may not only reduce LOC107052320 expression but also alter transcript structure in white‐feathered WC chickens.

## 3 DISCUSSION

The integration of high‐quality genome and pan‐genome graphs offers the most effective approach for establishing a comprehensive catalog of SVs with precise breakpoints and sequence content at population scales. This approach offers a significant opportunity to investigate the mechanisms underlying SV formation on a large scale, and understand their impacts on genetic structure, gene regulation, and complex traits in chickens. To accomplish this, we developed an integrated pipeline that generates haplotype‐resolved and systematically annotated assemblies of the WC chicken, a native breed in China. These new genome assemblies, containing representative genetic information for chickens in southern China, serve as a valuable supplement for identifying genetic variations among different chicken strains worldwide.

To enable precise SV genotyping, we constructed pan‐genome graphs by integrating 31 assemblies with a catalog of SVs obtained from 354 resequencing samples. However, since our assemblies used a male Wenchang chicken, they excluded the W chromosome, which exists only in females. Consequently, the graph‐based pangenome lacks W chromosome data, which limits our understanding of sex‐specific genetic elements. This approach yielded 185,205 high‐confidence SVs across diverse chicken strains and populations. These SVs serve as excellent resources for investigating their formation mechanisms, genetic characteristics, and functional implications. We observed that pangenome‐based SV detection is far superior to second‐generation sequencing methods, particularly in detecting large insertions. Our findings revealed that only one‐third of SV formation can be attributed to homology and TE insertion mechanisms. Unlike in mammals, where TE insertion is one of the key drivers of SVs, it only accounts for 10% of SVs in chickens. Additionally, TE insertion in chicken primarily involves a single LINE/CR1 family, accounting for nearly 90% of them. This discrepancy may arise from variances in transposon activity between chickens and mammals.

Using SVs as genetic markers, we found that the genetic diversity and geographic distribution patterns of Wenchang Chicken subpopulations closely aligned with SNP‐based analyses, with some variation between breeds. Two main factors explain this result: the distinct evolutionary roles of SVs in different breeds and the technical limitations in detecting SVs in certain genomic regions. These findings demonstrate the value of SVs as complementary makers for studying evolution and adaptation. It can be speculated that LD may be the main factor contributing to this similarity. To further investigate, we compared the genetic contribution of independent and SNP‐tagged SVs. The results showed that while independent SVs can have similar genetic components to SNP‐tagged SVs, they exhibit different characteristics. For instance, they may vary in terms of population frequency, average length, average counts, and IBS allele‐sharing. Our findings suggest that SVs carry genetic information that cannot be completely replaced by SNPs, especially independent SVs. The independent SVs reveal distinct genetic relationships between breeds, particularly showing increased genetic similarity between Red Junglefowls (RJFs) and commercial broilers. The distinctive patterns of these independent SVs, which are influenced by evolutionary pressures or genetic exchange, require further investigation. Identifying and utilizing these independent SVs is crucial for a comprehensive understanding of genetic diversity, including aspects such as missing heritability and introgression evidence.

Notably, SVs have been shown to be associated with regulatory elements. We found that SVs tend to concentrate more in active regions of the genome, regardless of their frequency or the specific tissues. However, when examining the association of SVs with the H3K27me3 histone mark, we observed a significant enrichment of high‐frequency SVs, particularly in the immune system (thymus, spleen, bursa, and bone marrow), respiratory system (lung and trachea), and muscle tissues. Since these tissues are associated with immunity, the suppressed regulation of high‐frequency SVs in them may be related to immunity‐driven adaptive selection during chicken evolution. For instance, high cellular turnover and pathogen‐driven selection pressure favor SVs in antigen presentation genes [56].

Our study identified 1728 potential molQTL‐SVs that are strongly linked with known molQTL‐SNPs. These SVs displayed unique frequency spectra across different strains and populations, with a particularly noticeable difference between broilers and layers. For instance, we observed an insertion in the exon of the *EEF1A2* gene, which is associated with egg‐laying rates. Furthermore, a molQTL‐insertion downstream of the *JPT2* gene showed a significant frequency difference between RJFs and domestic chickens, confirming the previous results [7]. These findings can enhance our understanding of how candidate genes might influence gene expression through SVs that may have been overlooked in previous studies.

To evaluate the potential impact of SVs on complex traits in chickens, we concentrated on feather traits associated with domestication and productivity. We found a VNTR‐mediated deletion in the exon of a lncRNA (LOC107052320). This lncRNA was prevalent in a recessive haplotype in white‐feathered chickens and exhibited significant differentiation compared to colored feathers. Importantly, the SV within the haplotype suggests gene flow from white layers to white‐feathered WC chickens. This discovery provides new insights into feather color formation and helps identify new markers for standardizing chicken breed definitions in breeding practices.

## 4 CONCLUSION

In summary, our findings reveal a diverse genetic landscape across different chicken strains, exhibiting a wide range of SVs. These results not only deepen our understanding of genetic diversity within the chicken populations but also provide insights into the characteristics and functions of SVs. Importantly, they underscore the importance of incorporating SVs in genetic investigations related to complex traits and chicken breeding.

## 5 METHODS

### Sample collection

Ten populations of WC chickens were selected for whole‐genome resequencing. Each population consists of 10 males and 10 females selected at random, with a total of 20 individuals per population. These populations are named after the breeding farm's abbreviation: CB, CK, CU3, LA, LD, TN, WB, WJ, WY, and WZ. Each individual was phenotypically characterized for body weight (g) at five time points and for feather color records. A male WC chicken with yellow feathers was selected for genome assembly, and samples were collected from its 23 tissues.

### Sample sequencing

For the resequencing of 200 samples, genomic DNA was extracted from chicken blood FTA card samples. Whole‐genome resequencing was performed using the MGI‐2000/MGI‐T7 platform at Shenzhen BGI Technology Co. Ltd. For the genome assembly, high‐molecular‐weight genomic DNA was prepared using the cetyltrimethylammonium bromide (CTAB) method and purified using the Qiagen genomic kit. This DNA was used to construct PacBio HiFi sequencing libraries (PacBio Sequel IIe) and ONT Ultra‐long sequencing libraries (PromethION with SQK‐LSK110 chemistry). The same individual was used to construct Hi‐C libraries and Illumina paired‐end short insert libraries (PE 150 bp) to generate chromosome‐scale genomes. Additionally, 23 tissue RNA libraries were generated for Illumina RNA‐seq to annotate the genome.

### Genome assembly

The genome size of WC chicken was estimated using Jellyfish [57] (v2.3.0) and GenomeScope [58] (v2.0). This estimation was based on k‐mers of length 21 obtained from Illumina paired‐end reads. The genome assembly pipeline followed the HG002 trio‐phased T2T assembly best practices [59] with custom modifications, including Hi‐C based binning, hybrid assembly, ONT assembly, Scaffolding, Gap filling, and Polishing, as depicted in Figure S1.

PacBio HiFi reads were processed to remove reads containing adapters using HiFiAdapterFilt [60] (v2.0.0) with the default parameters. Then, phased contig assemblies were generated by combining the HiFi reads and paired‐end Hi‐C reads using Hifiasm (v0.16.1) in Hi‐C mode [35]. The Illumina paired‐end reads and Hifi reads were assigned to two phased haplotypes based on unique k‐mers using the trio_binning program [61] (v1.0.0) (https://github.com/esrice/trio_binning).

Hybrid assembly genomes were assembled by combining the PacBio HiFi and ONT ultra‐long reads using Verkko [37] (v1.1). Before Verkko, the phased contig assemblies by Hifiasm were used to exclude read pairs containing k‐mers only found in the other haplotype using meryl [62] (v1.3). The ONT assembly was assembled using NextDenovo (v2.5.0) with the default parameters and polished with NextPolish [63] (v1.4.1) (https://github.com/Nextomics/) with three rounds of alignment with Illumina paired‐end reads.

The hybrid assemblies from Verkko underwent redundancy removal using purge_dups (v1.2.6) (https://github.com/dfguan/purge_dups). The resulting contigs were then anchored to the chromosome level using Salsa2 [64] (v2.3) and YaHS [65] (v1.2a), respectively, utilizing Hi‐C reads. The Juicebox assembly tools (v2.18) (https://github.com/aidenlab/Juicebox) were employed for manually correcting the connections. Subsequently, to increase the completeness, the anchored scaffolds were further scaffolded and gap‐closed using RagTag [66] (v2.1.0) and manual methods. Gap resolution was performed using both ONT assembly (25 gaps resolved in WChap1 and 42 in WChap2) and phased HiFi assembly (3 gaps resolved in each of WChap1 and WChap2). Finally, the assembled scaffolds were iteratively polished by HiFi reads using a repeat‐aware polishing strategy and by Illumina paired‐end reads using the freebayes‐polish strategy [67] with recommended parameters.

### Genome quality assessment

The assembly quality was evaluated by referring to the post‐evaluation of the vgp‐assembly pipeline [67] (https://github.com/VGP/vgp-assembly/tree/master/pipeline). Supporting evidence from PacBio HiFi reads, Hi‐C reads, and Illumina paired‐end reads was used to identify reliable regions of the phased assemblies using the Asset evaluation tool (v1.0.3) (https://github.com/dfguan/asset). The telomeric‐identifier (v0.2.63) (https://github.com/tolkit/telomeric-identifier) was employed to identify the telomere regions for phased assemblies. The telomeric sequence in chicken is typically represented as (TTAGGG)n. Genome completeness was assessed using the BUSCO program [68] (v5.4.3), which contains the 8338 single‐copy orthologs of the avers_odb10 database. Base accuracy was measured by assembly QV using Merqury [62] (v1.3), which compares the 21‐mers found in short reads and the assembly sequences.

### Genome annotation

Repetitive sequences in the phased assemblies were identified by integrating both *de novo* and homology‐based predictions. *De novo* prediction was performed using LongRepMarker in the *de novo* mode. Repeat libraries generated by LongRepMarker [69] (v2.0) were utilized to mask repetitive sequences of the phased assemblies using RepeatMasker [70] (v4.1.5). We annotated the coding regions of the phased assemblies using a strategy that combines four annotation methods: annotation liftover, ab initio predictions, RNA expression evidence, and protein homology search. 1) Gene annotations from GRCg7b (GCF_016699485.2) were lifted over to the WChap1 assembly using Liftoff [71] (v1.6.3). 2) *Ab initio* gene prediction based on phased genome sequences was performed using AUGUSTUS [72] (v3.5.0) and GeneMark‐ES [73] (v4.71). 3) Hisat2 [74] (v2.2.1) was adopted to map RNA‐seq reads to the genome. The mapped reads were then subjected to assemble transcripts with StringTie [75] (v2.2.1) and predict ORFs with TransDecoder (v5.5.0). Additionally, the BRAKER2 [76] (v2.1.6) pipeline was used to predict protein‐coding gene structures using the mapped reads. 4) Exonerate [77] (v2.3.0) and GenomeThreader [78] (v1.7.1) were employed for homology‐based gene prediction, using protein sequences from the NCBI (*n* = 68,683) and UniProtKB (*n* = 27,528) datasets.

EvidenceModeler [79] (v1.1.1) was used to integrate all the evidence above to predict the nonredundant gene annotation. The integration of the gene models mentioned above, as well as the identification of alternative splice sites and UTR annotation, was performed using the annotation pipeline PASA (v2.5.3). In addition, the gene annotation of phased assemblies was obtained from a well‐annotated reference genome (GCF_016699485.2) using the annotation liftover strategy [71]. The final gene annotations that were not present in the lifted gene annotation were merged with our annotation data set using GFF3toolkit. The completeness of the gene repertoire was evaluated using BUSCO (v5.4.3).

### SNP and InDel calling

Paired‐end 150 bp reads from 354 samples with high depth (>10×) were trimmed using fastp [80] (v0.23.2) with the parameters “‐q 20 ‐u 30 ‐l 75”. The clean reads for each sample were then aligned to the WChap1 genome using BWA [81] (v0.7.17) with the default parameters. PCR duplicate removal, read realignment, quality score recalibration, and SNP and InDel calling were performed using the Sentieon [82] software. Then, the GVCFtyper module of Sentieon was used to generate a merged VCF file that includes genotypes from multiple single‐sample GVCF files. For the initial quality control of variants, vcftools [83] (v0.1.16) and GATK [84] (v4.3.0) were used with different filtration criteria for SNPs and InDels, respectively. The following criteria were used for SNPs: 1) max‐missing 0.3; 2) max‐alleles 2; 3) maf 0.01; 4) min‐meanDP 3; 5) QD < 2.0; 6) QUAL < 30.0; 7) SOR > 3.0; 8) FS > 60.0; 9) MQ < 40.0; 10) MQRankSum < −12.5; and 11) ReadPosRankSum < −8.0. For InDels, the following criteria were used: 1) max‐missing 0.3; 2) max‐alleles 2; 3) maf 0.01; 4) min‐meanDP 3; 5) QD < 2.0; 6) SOR > 10.0; 7) QUAL < 30.0; 8) FS > 200.0; and 9) ReadPosRankSum < −20.0.

### SV calling using short reads

To perform SV calling using Illumina paired‐end reads, several algorithms are available, including Read‐pair (RP), Split‐read (SR), Read depth (RD), and Assembly (AS). In our study, we adopted an integrated approach that combines multiple SV detection algorithms to maximize sensitivity [18]. Specifically, we applied six detection tools, each with at least two algorithms: Manta [85] (v1.6.0), Delly [86] (v0.8.3), Wham [87] (v1.7.0), Smoove [88] (v0.2.8), Dysgu [89] (v1.4.0), and GRIDSS2 [90] (v2.13.2). We tracked SV coordinates per sample for each tool using SURVIVOR (v1.0.7) with parameters 50 1 1 1 0 50 and filtered them using parameters NA 50 100000 0 −1. We then merged the results by variant type, as different tools supported different SV types—all six software tools supported deletions, while only three tools (Dysgu, Manta, and Wham) supported insertions. After merging SV coordinates from all samples and tools, we calculated coordinate frequencies. We used bedtools intersect to identify the overlapping SV clusters. For each cluster, we selected the most frequent coordinates across all samples and software as representative coordinates. For deletions, we determined the SV sequence using these representative coordinates and genome sequences. For insertions, we extracted SV sequence information from Manta.

### Pangenome construction from genome alignments

We utilized published assemblies of all 30 samples from NCBI (Table S1), in addition to our new assemblies, to detect SVs based on using the pangenome variation graph assembly. This was achieved using Pggb [39] (v0.5.3) with parameters ‐p 95 ‐s 10000 ‐T 20 ‐‐poa‐params 1,9,16,2,41,1. Next, we called variants on the WChap1 assembly of all autosomes and chromosome Z using the vg toolkit (v1.40.0) in deconstruct mode with default parameters. The resulting VCF file contained variant calls made across all 31 other assemblies.

### Pan‐genome graph construction

The read‐based SV and assembly‐based SVs from pangenome were further merged by their coordinates. If there were conflicting overlaps in coordinate information, we retained the assembly‐based SVs. Next, The WChap1 assembly served as the backbone of the pan‐genome graph. All SVs previously identified were incorporated into a variant graph using the “construct” module of the vg toolkit without removing any alternate alleles. The resulting pan‐genome graph was then indexed in XG and GCSA formats using “vg index,” with the “‐L” parameter enabled for both formats. SVs were depicted as bubbles in the graph, with paths representing the corresponding alleles. These paths included the start and end nodes of reference sequences, as well as the paths traversing these nodes.

### Graph‐based SV genotyping

A total of 354 high‐depth (>10×) samples were genotyped using the pan‐genome graph. The clean reads for each sample were mapped against the graph genome using vg Giraffe [41], resulting in alignments in the GAM format. Alignments with a mapping quality <5 or base quality <5 were excluded. Subsequently, a compressed coverage index was calculated using “vg pack”, and snarls were generated using “vg snarls,” both with default parameters. SV genotyping results for each of the 354 samples were produced using “vg call” with the parameter “‐v ‐‐bias‐mode ‐‐het‐bias 2,4” on the constructed pan‐genome graph.

### Genomic variation annotation

We used ANNOVAR [91] (v20221005) for gene‐based annotations of the identified SVs, InDels, and SNPs. The WChap1 genome annotation was used to provide information on exonic regions, splicing sites, intronic regions, 5′ and 3′ untranslated regions (UTRs), upstream and downstream regions, and intergenic regions.

### Breakpoint analysis

We conducted a detailed mechanism analysis for all SVs, including deletions and insertions, with precise breakpoints, using the BreakSeq pipeline [46, 92]. This pipeline utilized distinct sequence‐based signatures located within and around the breakpoint junction of each specific SV. The five categorized mechanisms are as follows: (1) VNTR (Variable Number Tandem Repeat): The repeatMasker program identified extensive coverage of tandem repeats and low‐complexity regions within a given SV sequence, covering 30% of SV regions. (2) NAHR (Non‐Allelic Homologous Recombination): Extensive homology of breakpoint junctions was used to identify NAHR events. For SVs greater than 1 kb, the breakpoint junctions were set to 1 kb in length, with a flanking sequence length of 1 kb and a homology length cutoff of ≥200 bp. For SVs less than 1 kb, the breakpoint junctions are set to 50 bp in length, with a flanking sequence length of 200 bp and a homology length cutoff of ≥20 bp. (3) STEI (Single Transposable Element Insertion) and MTEI (Multiple Transposable Element Insertions): SV sequences were considered TEIs if they aligned to known interspersed mobile element insertions (MEIs) in the genome and covered more than 30% of SV regions. These were further subclassified into STEI or MTEI based on whether the SV was aligned to a single or multiple transposable element insertion(s). (4) NHR (Nonhomologous Recombination): Sequences lacking any of the above‐mentioned signatures were classified under the nonhomologous recombination mechanism class.

For the deletion SV in the exon of the LOC107052320 lncRNA locus, we employed TRF (V409. linux64) for identification of a 430 bp tandem repeat unit, utilizing the following parameters: “2 7 7 80 10 50 500 ‐m ‐f ‐d.” We then performed alignments using blastn (V 2.5.0) to determine the copy number and breakpoints for each unit.

### Population genetics analysis

Before constructing the phylogenetic tree, the SNP data set was pruned using PLINK [93] (v1.90). The following parameters were used: “‐‐maf 0.1 ‐‐indep ‐‐pairwise 500 50 0.2”. This pruning was based on an LD threshold of 0.2 and *a minor* allele frequency greater than 0.1. Next, we converted the SNPs from VCF format to phylip format using the vcf2phy [94] (v2.0). Finally, the neighbor‐joining tree was constructed (bootstrap = 100) using the Phylip tool [95] (v3.697).

Unsupervised admixture analysis on SNPs was conducted using ADMIXTURE [96] (v1.3.0). The analysis was run with *K* = 2 to *K* = 16, and the corresponding cross‐validation errors (CV) were calculated. Population genetic structure was assessed through PCA using whole‐genome SVs and SNPs with Plink (v1.90) and the “‐‐pca” parameters. Additionally, the UMAP algorithm in R was utilized to evaluate the clustering among SVs based on their population frequency in various strains.

LD decay was calculated using PopLDdecay [97] (v3.42) with default parameters. The pairwise *R* ^2^ values were estimated with the default maximum distance and averaged across the entire genome. The LD for each strain was calculated using SNP pairs specific to that strain.

Runs of homozygosity in each strain were estimated using plink (v1.90) with parameters “‐‐homozyg‐window‐snp 50 ‐‐homozyg‐snp 50 ‐‐homozyg‐kb 300 ‐‐homozyg‐density 50 ‐‐homozyg‐gap 100 ‐‐homozyg‐window‐missing 5 ‐‐homozyg‐window‐threshold 0.05 ‐‐homozyg‐window‐het 3.”

Identity‐by‐descent (IBD) blocks in the whole genome between each sample were estimated using the hap‐ibd [98] (v1.0) software. nIBD was calculated by transferring the IBD region from paired individuals to paired strains, defined as cIBD/tIBD, where cIBD represents the count of all haplotypes IBD between strain A and strain B, and tIBD represents the total pairwise comparisons between strain A and strain B [99].

### Strain‐based NJ tree construction

SVs having strong or weak linkage with SNPs (*R* ^2^ ≥ 0.2 or <0.2) were defined as SVs with or without LD, respectively. To explore the strain‐based genetic distance, various types of variations, including SNPs, SVs with LD, and SVs without LD, were used to construct the NJ tree. The IBS distance matrix of 354 samples was calculated using Plink (v1.90) with parameters “‐‐distance‐matrix, maf 0.05.” Then, the distance matrix was converted to strain levels to represent genetic differentiation between each pairwise population. For example, to calculate the average IBS distance between strain A and strain B, we determined the average IBS distance among all combinations of individuals within strain A and individuals within strain B. Finally, the neighbor‐joining tree was constructed using FastME [100] (v2.0) with default parameters and visualized using iTOL (https://itol.embl.de/) with default settings.

### Regulatory element and 3D chromatin annotation sources

Regulatory element annotations used in this study correspond to seven types of cis‐regulatory elements (cCREs) from the Chicken FAANG and four types of molQTLs from the ChickenGTEx (http://chickengtex.ic4r.org/download). To match our variation data, we mapped all genomic coordinates of regulatory element annotations in galGal6 to the WChap1 assembly using Liftoff [71] (v1.6.3). The chain file used by LiftOver was generated by the transanno tool in the minimap2chain module.

The A/B compartments were identified using HiCExplorer [101] (v2.1). The process involved converting paired‐end Hi‐C reads into a normalized “.cool” matrix with 100‐kb bin resolution using Knight–Ruiz (KR) normalization. Then, PCA was performed via the hicPCA module to derive the first eigenvector for compartmentalization.

### Feature enrichment of genomic regions

To evaluate the enrichment level of specific elements in different genomic regions, we compared the actual number of overlapping elements with a specific region to the average value obtained by randomly assigning the same number of elements. When regulatory elements overlap with SV by more than 1 base pair (bp), they are defined as SV‐overlapping elements. In this study, the enrichment *Z*‐scores of genomic regions for different SV catalogs were calculated using permutation tests implemented in the regionR package [102], with the number of permutations set to 100.

### GWAS analysis

Phenotype data for body weight traits were collected from 200 chickens across five different time periods. The GWASs were performed using a linear mixed model implemented in GEMMA [103] (v1.0.3), with adjustments made for gender, kinship, and population structure as cofactors. The kinship matrix was computed using all the SNPs in GEMMA, and the population structure was determined using the top 10 principal components. The genome‐wide significance threshold was determined using a uniform threshold of 0.05/*n*, where *n* represents the effective number of independent SVs and SNPs calculated using the Genetic Type I error calculator. In addition, to compare WC chickens with colored feathers and white feathers, we performed a GWAS on the SV data set. This was done using a case‐control study design and logistic regression model in the Plink (v1.90) software.

### Selection scan for white feathers

Selective sweeps across the WC populations between the four colored feathers and white feathers were identified using genome‐wide SNPs. We employed three methods with a sliding window of 30 Kb with a step size of 10 Kb. These methods include calculating the genetic differentiation (*F*_*ST*_) by vcftools (v0.1.16) tool, the cross‐population composite likelihood ratio test (XP‐CLR) by xpclr [104] (v1.1.2) software, the Cross Population Extended Haplotype Homozygosity (XP‐EHH) by xpehh [105] (v1.3.0) software.

To effectively combine multiple selection signals, we applied the following strategy. Taking *F*_ST_ as an example, we converted the *F*_ST_ sweep signals within each bin (30 Kb) into rank values. The variable *F*_ST_ was ranked and normalized to obtain values *F*_ST_R ranging from 0 to 1. Next, *F*_ST_R was scaled to *F*_ST_S using a factor to prevent division by zero (1.001 was added to the denominator). The formula used was: *F*_ST_S = *F*_ST_R/(1.001 − *F*_ST_R). The final rank values were calculated by averaging *F*_ST_S, XP‐CLRS, and XP‐EHHS. The rank values falling in the top 0.1% were considered potential genomic regions under selection. The CMplot R package (https://github.com/YinLiLin/CMplot) was then used to generate a Manhattan plot displaying the rank values for each bin.

### Linkage disequilibrium (LD) block analysis

The genotype data for WC chickens with white and colored feathers were processed using vcftools. The filtered data was then analyzed with LDBlockShow [106] (v1.40). The LD blocks were plotted to illustrate the difference in LD blocks between WC chickens with white and colored feathers.

### ABBA‐BABA statistics in sliding windows

To evaluate evidence of gene flow between chicken populations, we computed the D statistic and f estimators across the genome using ABBABABAwindows.py scripts from the genomics_general tools (https://github.com/simonhmartin/genomics_general). The model for ABBA‐BABA statistics was constructed using the relationship (((P1,P2),P3),O), where RJFt was the outgroup O, WC chickens with different feather colors were P1 and P2, and WL and BL were selected as P3, the control group, to test the significance of gene flow.

### SV PCR validation

Genomic DNA was extracted from frozen blood, followed by PCR performed with 2× Blood Direct PCR MasterMix (TIAN GEN). The SV was analyzed via genotyping PCR using primer pairs: 5′‐TACGCCAACCAAGTCACCAG‐3′ and 5′‐GTGAAGGGAAGAGACCTGGG‐3′. These were designed with Primer‐BLAST for amplification of both reference and alternate alleles. The final reaction consisted of a total volume of 25 μL, which included 1.25 μL blood template, 0.625 μL forward primer, 0.625 μL reverse primer, 12.5 μL 2× Blood Direct PCR MasterMix, and 10 μL RNase‐Free ddH2O. PCR thermocycling was as follows: initiation at 95°C for 3 min, denaturation at 95°C for 15 s, annealing at 54°C for 20 s, and extension at 72°C for 30 s. This cycle was repeated 30 times, with a final extension at 72°C for 5 min.

### qRT‐PCR validation of SV

We collected testis samples from the same batch of young and middle‐aged white and yellow roosters, following aseptic procedures. These samples were quickly frozen in liquid nitrogen and stored at −80°C in an ultra‐low temperature refrigerator for RNA extraction, cDNA reverse transcription, and qRT‐PCR analysis (all using TIAN GEN kits).

Before validating lncRNA qPCR, the individual's SV homozygosity was verified. Primer pairs were designed using the previously mentioned PCR validation method. DNA was extracted from frozen testes using these primer pairs: 5′‐CTCCCTACGCCAACCAAGTC‐3′ and 5′‐ACGTGGGTATGTCACACCAG‐3′. All samples confirmed to be homozygous were then used for qRT‐PCR quantification (Figure S10).

The qRT‐PCR reaction system included SYBR Green qPCR Mix 10 µL and 0.5 µL specific primers for each gene (5′‐TGACAGCTCAGACCAGAGGA‐3′ and 5′‐CTACGCCAACCAAGTCACCA‐3′). The thermal cycle parameters were as follows: predenaturation at 95°C for 5 min in one cycle, denaturation at 95°C for 10 s, 60°C for 30 s, and 60°C for 30 s over 40 cycles for annealing, extension, and data collection. After amplification, the melting curves were analyzed and stored at 95°C for 5 s, 65°C for 1 min, 97°C for 1 s, and 4°C. We repeated the qRT‐PCR analysis three times for each sample. The mRNA expression of the Lnc gene in white and yellow feathers was quantified by repeating each sample 3 times. The mean threshold period (CT) value was calculated for each sample gene in each sample. The relative expression of each gene was determined by the 2^−ΔΔCt^ method.

### Statistical analysis

The two‐sided Wilcoxon test was used to assess three types of statistical significance: the differences in frequencies and lengths between independent SVs and SNP‐tagged SVs, the fdM distribution for each expected model when P3 falls between WL and BL, and the qRT‐PCR results for LOC107052320 in WC chickens with white and yellow feathers.

## AUTHOR CONTRIBUTIONS

**Lihong Gu**: Conceptualization; methodology; formal analysis. **Chen Peng**: Software. **Anhong Chen**: Software. **Kaiyu Chen**: Software. **Xinli Zheng**: Data curation. **Dongyou Yu**: Data curation; writing—review and editing. **Zhengguang Wang**: Data curation; writing—review and editing. **Lingzhao Fang**: Conceptualization; writing—review and editing. **George E Liu**: Conceptualization; writing—review and editing. **Pengju Zhao**: Conceptualization; methodology; formal analysis; writing—original draft; writing—review and editing.

## CONFLICT OF INTEREST STATEMENT

The authors declare no conflicts of interest.

## ETHICS STATEMENT

Our study has been approved by the Animal Care and Use Committee of the Experimental Animal Center of Hainan Academy of Agricultural Sciences (HNXMSY‐20210533) and was conducted following the Regulations on the Management of Experimental Animals of the Ministry of Science and Technology of China (revised in March 2017).

## Supporting information

Supplementary Data V1.

**Figure S1.** Genome survey of the Wenchang chicken genome.
**Figure S2.** The pipeline for haplotype‐resolved genome assembly of the Wenchang chicken genome.
**Figure S3.** The use of various sequencing technologies ensured reliable blocks in the assemblies.
**Figure S4.** The pipeline for genome annotation of the Wenchang chicken genome.
**Figure S5.** The SV size distribution plot of deletions and insertions.
**Figure S6.** The distribution of coverage between six types of TE subfamilies and their associated SVs.
**Figure S7.** LD decay analysis for various chicken strains.
**Figure S8.** Frequency spectra of SVs for different chicken strains.
**Figure S9.** The overlap of molQTL SVs among different molecular phenotypes.
**Figure S10.** Assessment of homozygosity in SV of LOC107052320 for individuals to be verified through PCR validation.
**Table S1.** Telomere annotation for WChap1 and WChap2 genomes.
**Table S2.** TE annotation for WChap1 and WChap2 genomes.
**Table S3.** BUSCO results for different genome annotation strategies.
**Table S4.** The assembly accessions for the published genomes.
**Table S5.** Basic information for each chicken population.
**Table S6.** Body weight phenotypic information across chicken populations.

## Data Availability

Genome assemblies of the WC chicken have been deposited in the NCBI GenBank under the accession numbers PRJNA1063538 and PRJNA1063539. The annotations for the genome assemblies are available in the online resource of figshare (https://doi.org/10.6084/m9.figshare.24981066). Raw sequencing data for PacBio HIFI reads, ultralong ONT reads, Hi‐C reads, Illumina short reads, and 23 tissue RNA‐seq reads have been deposited in the National Genomics Data Center (NGDC) BioProject database (https://www.cncb.ac.cn/). The data can be accessed using the accession number PRJCA021177. All raw resequencing data of WC chickens have been deposited into the NCBI under study accession number PRJNA1047735. Genome assemblies of the WC chicken have been deposited in the NCBI GenBank under the accession numbers PRJNA1063538 (https://www.ncbi.nlm.nih.gov/bioproject/PRJNA1063538/) and PRJNA1063539 (https://www.ncbi.nlm.nih.gov/bioproject/PRJNA1063539/). The annotations for the genome assemblies are available in the online resource of figshare (https://doi.org/10.6084/m9.figshare.24981066). Raw sequencing data for PacBio HIFI reads, ultralong ONT reads, Hi‐C reads, Illumina short reads, and 23 tissue RNA‐seq reads have been deposited in the National Genomics Data Center (NGDC) BioProject database (https://www.cncb.ac.cn/). The data can be accessed using the accession number PRJCA021177 (https://ngdc.cncb.ac.cn/bioproject/browse/PRJCA021177). All raw resequencing data of WC chickens have been deposited into the NCBI under study accession number PRJNA1047735 (https://www.ncbi.nlm.nih.gov/bioproject/PRJNA1047735/). All software and their respective versions used in the study are publicly available as described in the Methods section. The pipeline for genome assembly, annotation, pangenome construction, SNP and InDel calling, and SV genotyping is available at https://github.com/PengjuZ/ChickenSV. The accessions for the previously published genomes can be found in Table S1. This study used published resequencing data from 154 samples, which were downloaded from the European Nucleotide Archive (ENA) with the accession number PRJEB30270 (https://www.ncbi.nlm.nih.gov/bioproject/PRJEB30270/). Supplementary materials (figures, tables, graphical abstract, slides, videos, Chinese translated version and update materials) may be found in the online DOI or iMeta Science http://www.imeta.science/imetaomics/.
