# Supplementary material for: Haplotype‐resolved genome and pan‐genome graphs reveal the impacts of structural variation on functional genome and feather colors in chickens
Source: IMetaOmics. 2025 May 24;2(3):e70027. doi: 10.1002/imo2.70027 (PMC12806048; doi:10.1002/imo2.70027)
Supplement: Supplementary file 2 — Figure S1. Genome survey of the Wenchang chicken genome. Figure S2. The pipeline for haplotype‐resolved genome assembly of the Wenchang chicken genome. Figure S3. The use of various sequencing technologies ensured reliable blocks in the assemblies. Figure S4. The pipeline for genome annotation of the Wenchang chicken genome. Figure S5. The SV size distribution plot of deletions and insertions. Figure S6. The distribution of coverage between six types of TE subfamilies and their associated SVs. Figure S7. LD decay analysis for various chicken strains. Figure S8. Frequency spectra of SVs for different chicken strains. Figure S9. The overlap of molQTL SVs among different molecular phenotypes. Figure S10. Assessment of homozygosity in SV of LOC107052320 for individuals to be verified through PCR validation. Table S1. Telomere annotation for WChap1 and WChap2 genomes. Table S2. TE annotation for WChap1 and WChap2 genomes. Table S3. BUSCO results for different genome annotation strategies. Table S4. The assembly accessions for the published genomes. Table S5. Basic information for each chicken population. Table S6. Body weight phenotypic information across chicken populations. [file IMO2-2-e70027-s002.docx]

**Supporting information to Haplotype-Resolved Genome and Pan-Genome Graphs Reveal the Impacts of Structural Variation on Functional Genome and Feather Colors in Chicken**

**Running title**: Pan-Genome Graphs Reveal SV Roles in Chicken Biology

Lihong Gu^1^, Chen Peng^2,3^, Anhong Chen^1^, Kaiyu Chen^2,3^, Xinli Zheng^1^, Dongyou Yu^2,3^, Zhengguang Wang^2,3^, Lingzhao Fang^4*^, George E. Liu^5*^, Pengju Zhao^2*^

^1^Institute of Animal Science & Veterinary Medicine, Hainan Academy of Agricultural Sciences, Haikou 570100, China.

^2^Hainan Institute, Zhejiang University, Yongyou Industry Park, Yazhou Bay Sci-Tech City, Sanya 572000, China.

^3^College of Animal Sciences, Zhejiang University, Hangzhou, Zhejiang 310058, China.

^4^Center for Quantitative Genetics and Genomics, Aarhus University, Aarhus, 8000, Denmark.

^5^Animal Genomics and Improvement Laboratory, Beltsville Agricultural Research Center, Agricultural Research Service, USDA, Beltsville, Maryland 20705, USA.

^*^Correspondence: [zhaopengju2014@gmail.com](mailto:zhaopengju2014@gmail.com) (Pengju Zhao); [george.liu@usda.gov](mailto:george.liu@usda.gov) (George E. Liu); [lingzhao.fang@qgg.au.dk](mailto:lingzhao.fang@qgg.au.dk) (Lingzhao Fang)

**Supplementary information**

**Figure S1.** Genome survey of the Wenchang chicken genome.

**Figure S2.** The pipeline for haplotype-resolved genome assembly of the Wenchang chicken genome.

**Figure S3.** The use of various sequencing technologies ensured reliable blocks in the assemblies.

**Figure S4.** The pipeline for genome annotation of the Wenchang chicken genome.

**Figure S5.** The SV size distribution plot of deletions and insertions..

**Figure S6.** The distribution of coverage between six types of TE subfamilies and their associated SVs.

**Figure S7.** LD decay analysis for various chicken strains.

**Figure S8.** Frequency spectra of SVs for different chicken strains.

**Figure S9.** The overlap of molQTL SVs among different molecular phenotypes.

**Figure S10.** Assessment of homozygosity in SV of LOC107052320 for individuals to be verified through PCR validation.

**Table S1.** Telomere annotation for WChap1 and WChap2 genomes.

**Table S2.** TE annotation for WChap1 and WChap2 genomes.

**Table S3.** BUSCO results for different genome annotation strategies.

**Table S4.** The assembly accessions for the published genomes.

**Table S5.** Basic information for each chicken population.

**Table S6.** Body weight phenotypic information across chicken populations.

**Supplementary Figures**

**
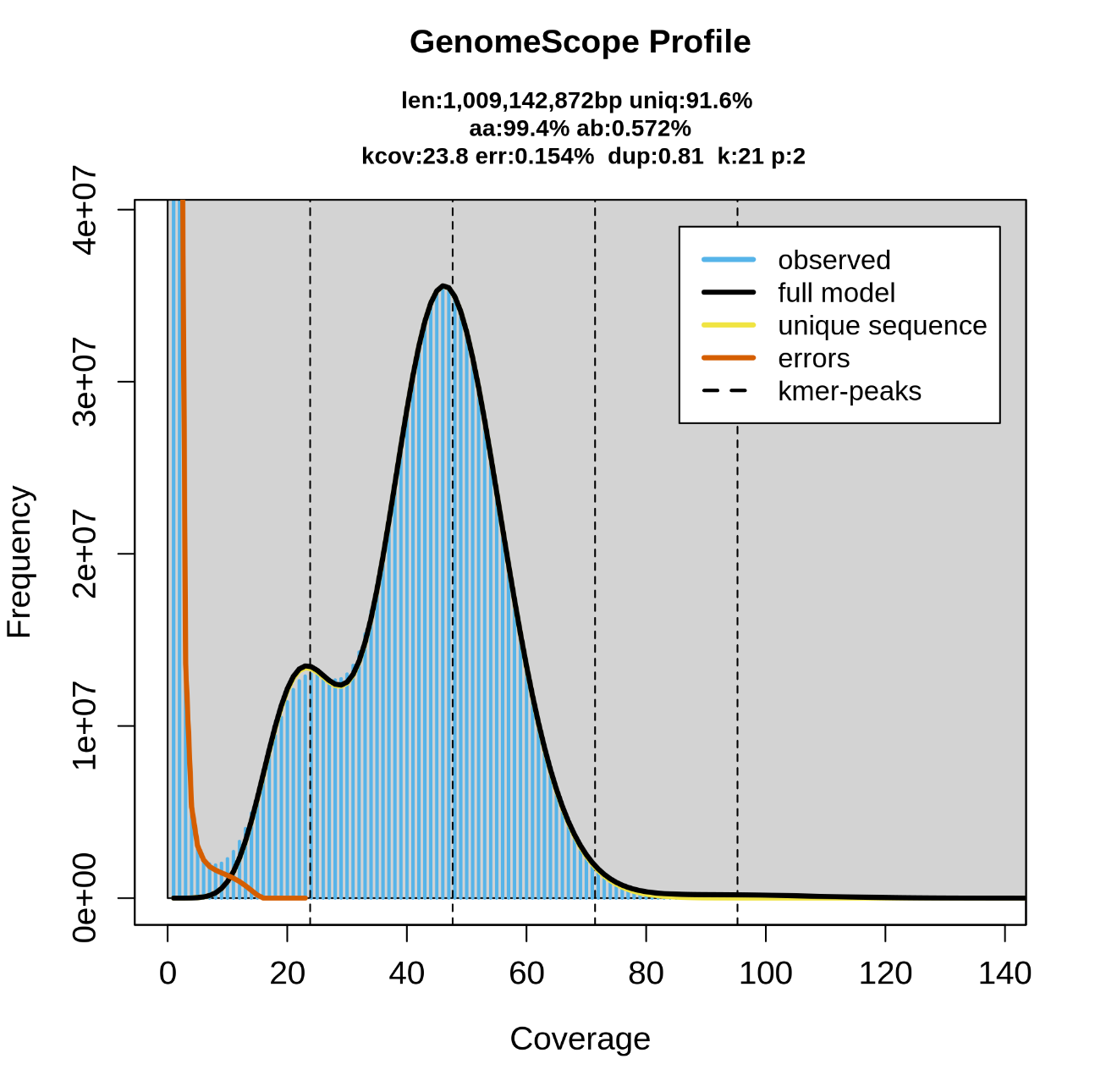
**

**Figure S1.** Genome survey of the Wenchang chicken genome.


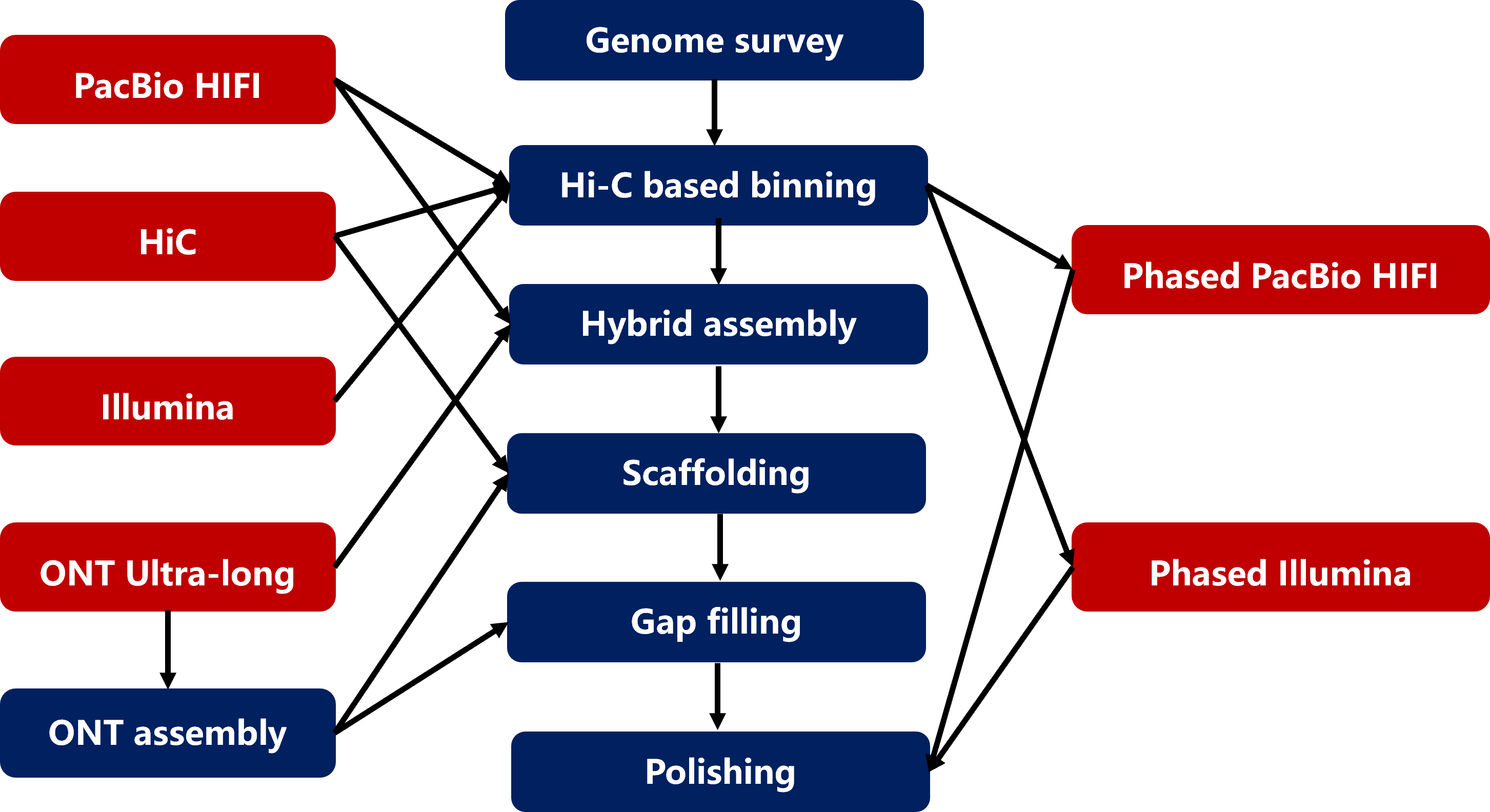


**Figure S2.** The pipeline for haplotype-resolved genome assembly of the Wenchang chicken genome.


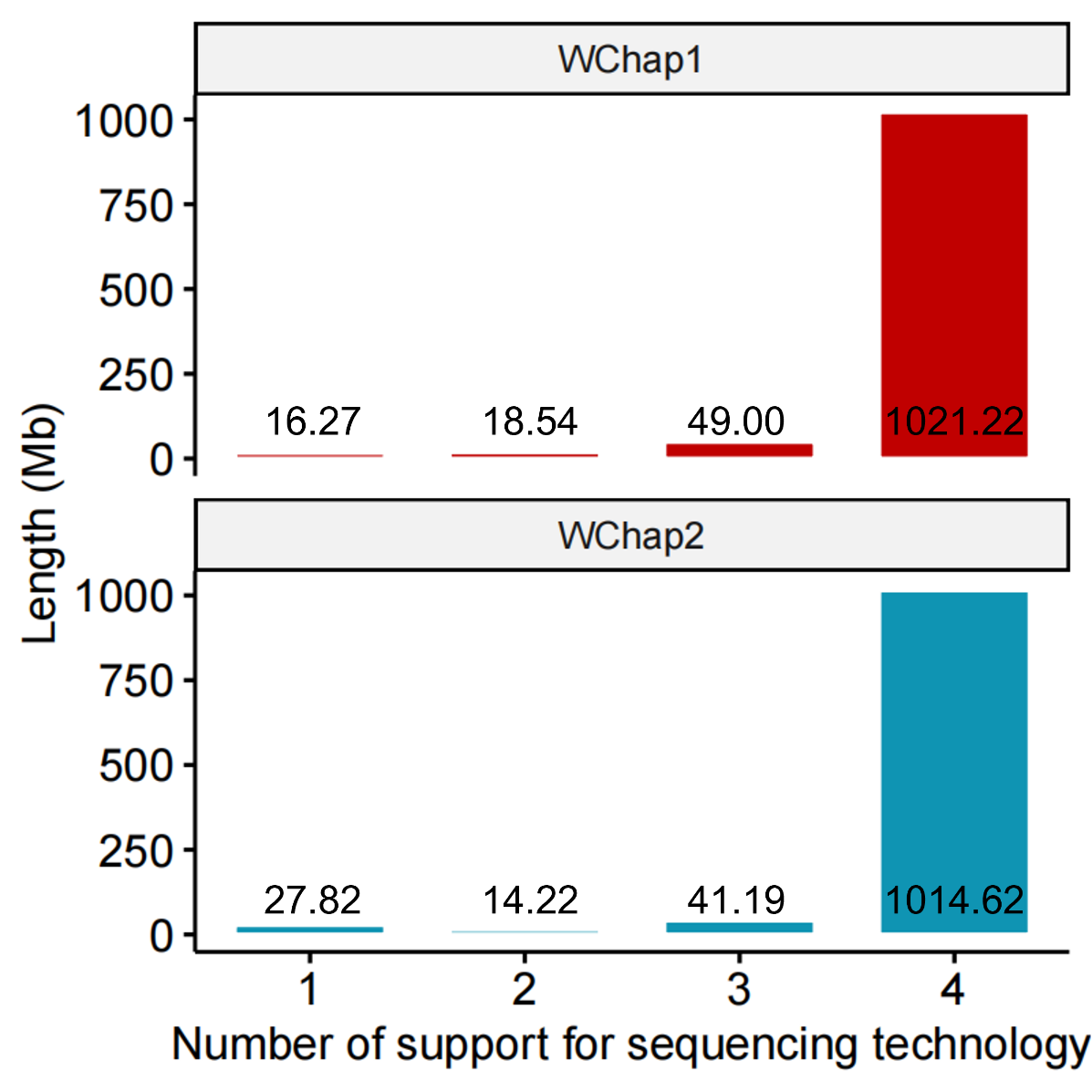


**Figure S3.** The use of various sequencing technologies ensured reliable blocks in the assemblies.


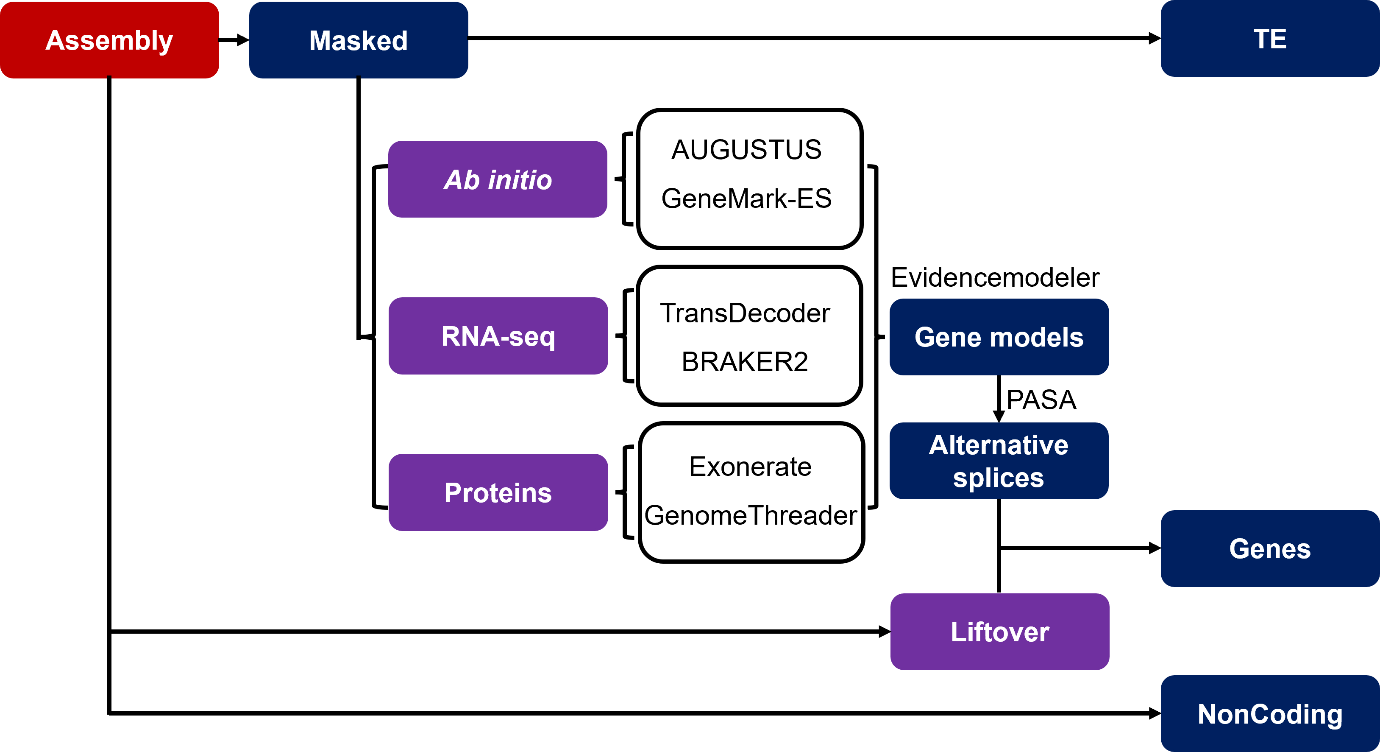


**Figure S4.** The pipeline for genome annotation of the Wenchang chicken genome.


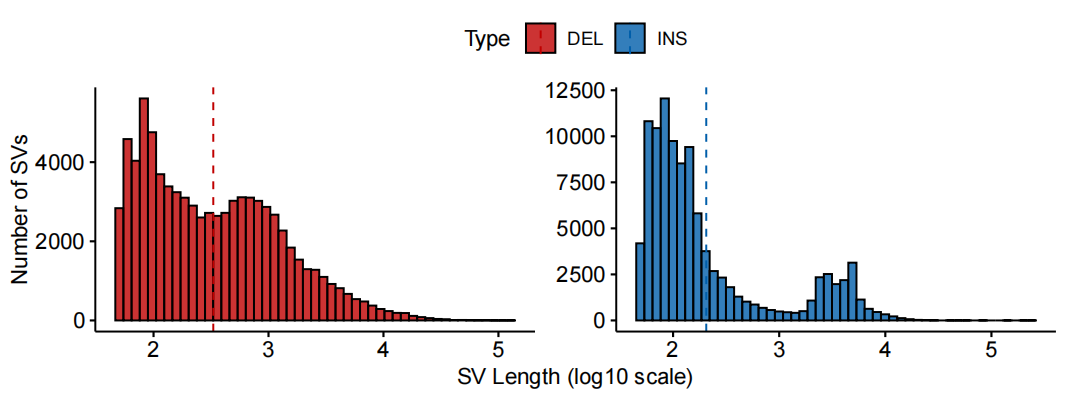


**Figure S5.** The SV size distribution plot of deletions and insertions. DEL: deletion, INS:insertion.


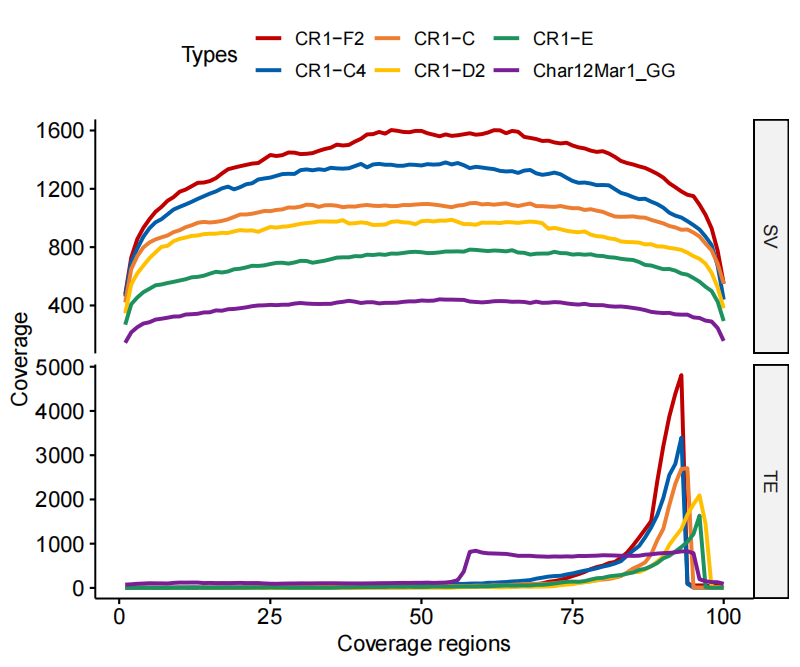


**Figure S6.** The distribution of coverage between six types of TE subfamilies and their associated SVs. The x-axis represents the overlapping proportion between SV and TE regions (as percentage), while the y-axis indicates the depth of SVs or TEs covered by the corresponding element.


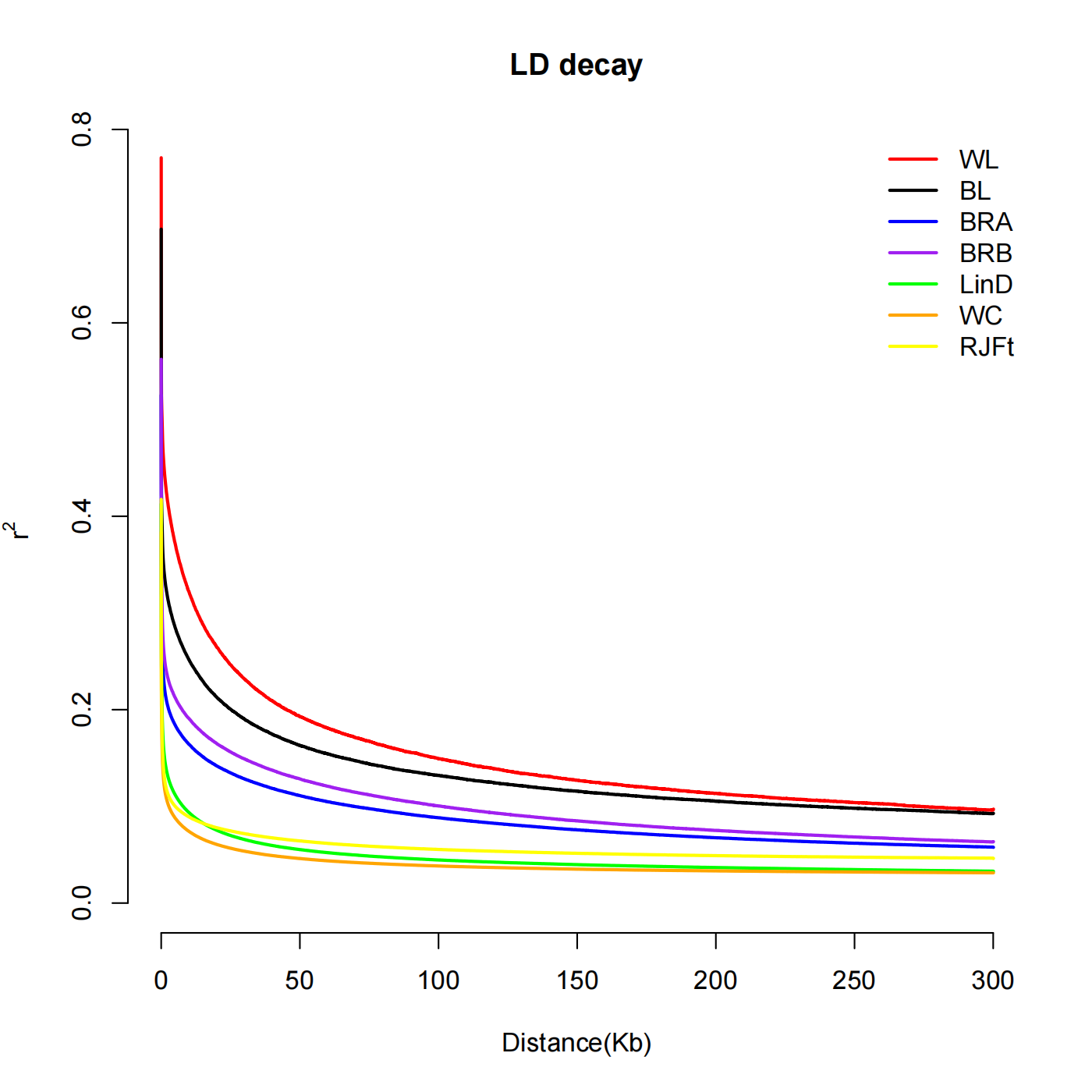


**Figure S7.** Linkage disequilibrium (LD) decay analysis for various chicken strains. The study encompasses several distinct populations: White layer (WL), Brown layer (BL), BRA and BRB Broiler lines (BRs), Lindian chicken (LinD), Wenchang chicken (WC), and red jungle fowl specimens from Thailand (RJFt).

**
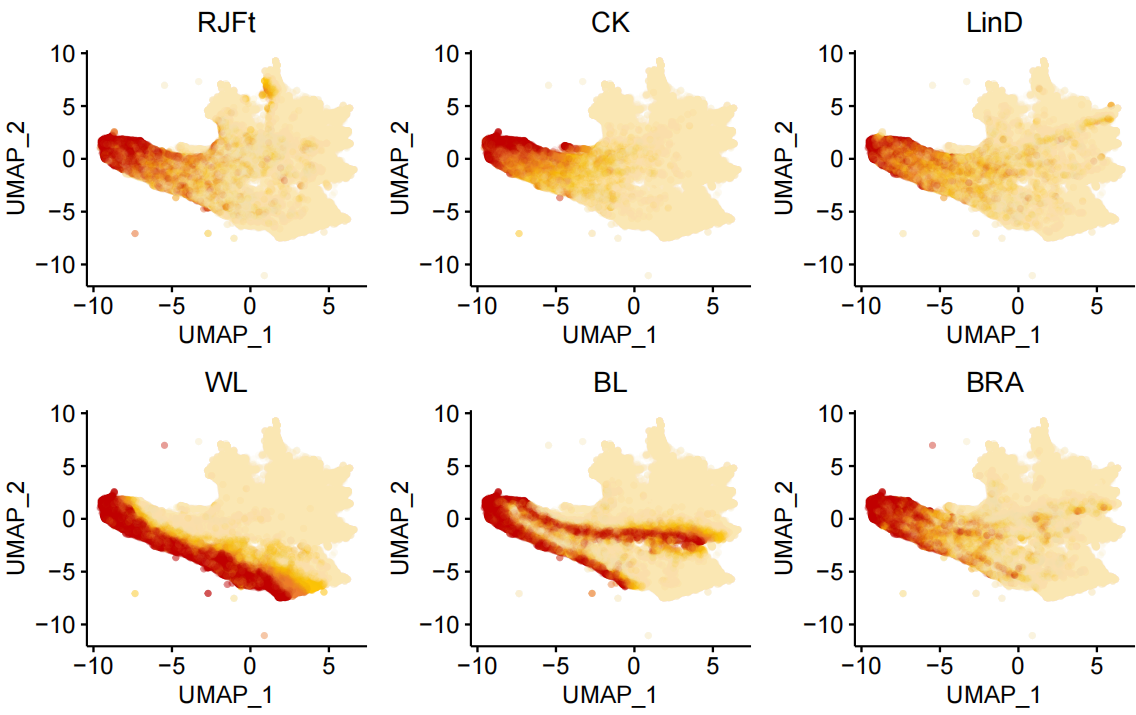
**

**Figure S8.** Frequency spectra of SVs for different chicken strains. One point in the figure represents one SV. The study encompasses several distinct populations: White layer (WL), Brown layer (BL), BRA Broiler lines (BRs), Lindian chicken (LinD), CK population in Wenchang chicken, and red jungle fowl specimens from Thailand (RJFt).


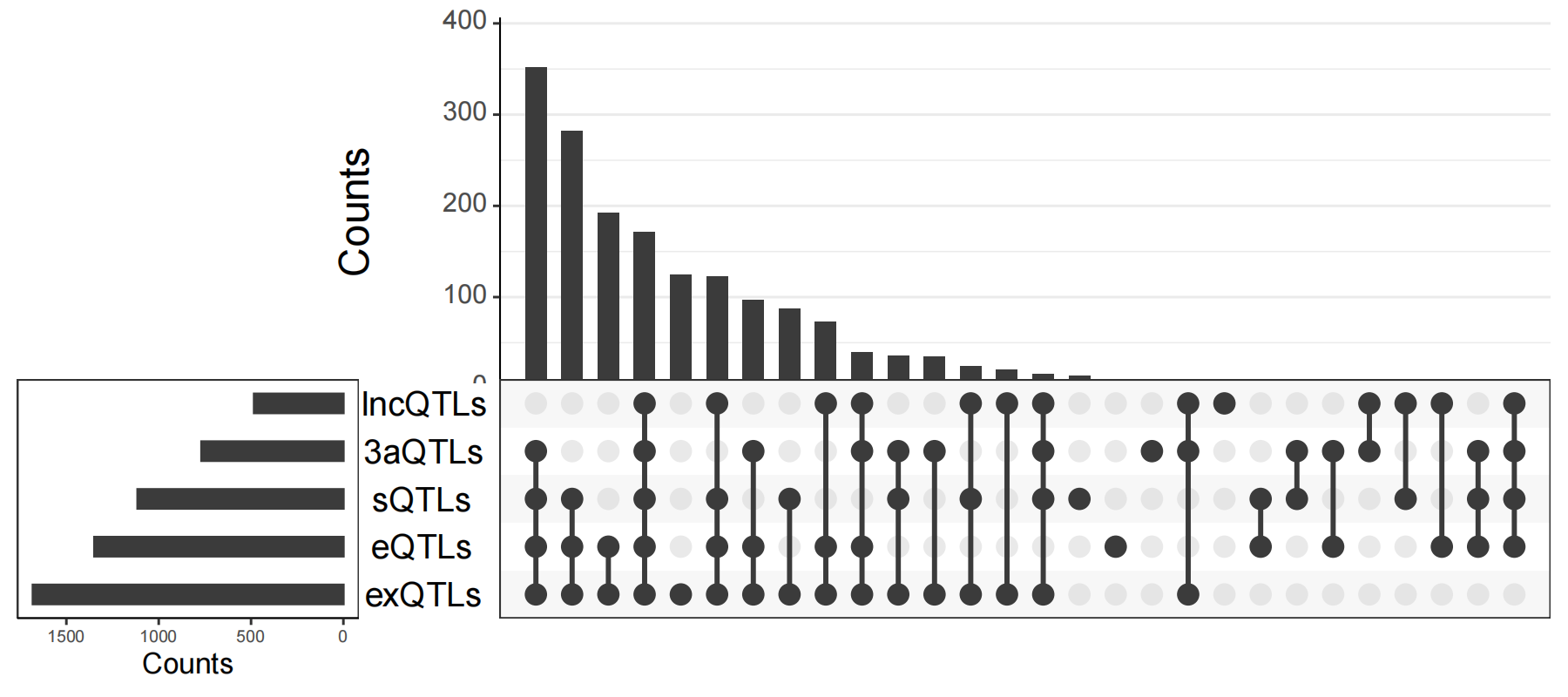


**Figure S9.** The overlap of molQTL SVs among different molecular phenotypes.


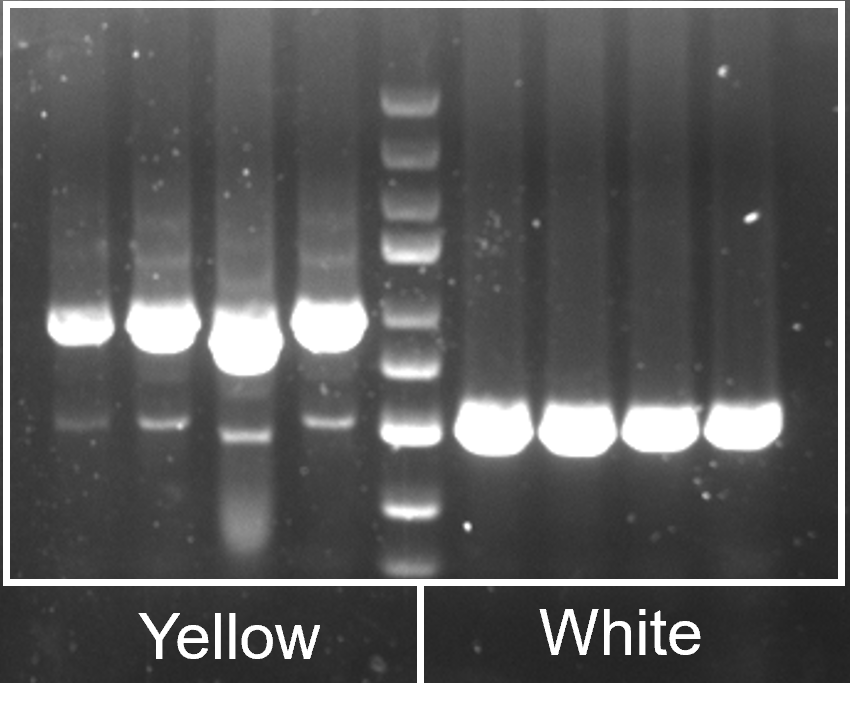


**Figure S10.** Assessment of homozygosity in SV of LOC107052320 for individuals to be verified through PCR validation.
